# Controlling multiple orderings in metal thiocyanate molecular perovskites A_*x*_{Ni[Bi(SCN)_6_]}†‡

**DOI:** 10.1039/d0sc06619b

**Published:** 2021-01-15

**Authors:** Jie Yie Lee, Sanliang Ling, Stephen P. Argent, Mark S. Senn, Laura Cañadillas-Delgado, Matthew J. Cliffe

**Affiliations:** School of Chemistry, University of Nottingham University Park Nottingham NG7 2RD UK matthew.cliffe@nottingham.ac.uk; Advanced Materials Research Group, Faculty of Engineering, University of Nottingham University Park Nottingham NG7 2RD UK; Department of Chemistry, University of Warwick Gibbet Hill Coventry CV4 7AL UK; Institut Laue Langevin 71 Avenue des Martyrs – CS 20156 38042 Grenoble France

## Abstract

We report four new A-site vacancy ordered thiocyanate double double perovskites, 
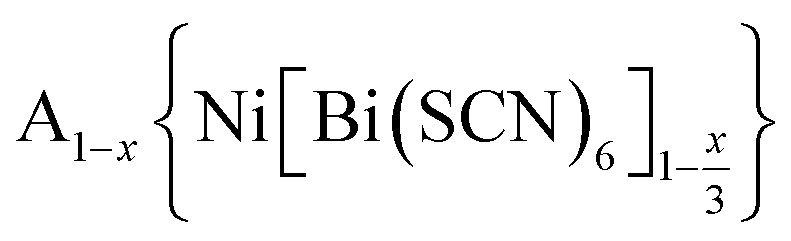
, A = K^+^, NH_4_^+^, CH_3_(NH_3_)^+^ (MeNH_3_^+^) and C(NH_2_)_3_^+^ (Gua^+^), including the first examples of thiocyanate perovskites containing organic A-site cations. We show, using a combination of X-ray and neutron diffraction, that the structure of these frameworks depends on the A-site cation, and that these frameworks possess complex vacancy-ordering patterns and cooperative octahedral tilts distinctly different from atomic perovskites. Density functional theory calculations uncover the energetic origin of these complex orders and allow us to propose a simple rule to predict favoured A-site cation orderings for a given tilt sequence. We use these insights, in combination with symmetry mode analyses, to show that these complex orders suggest a new route to non-centrosymmetric perovskites, and mean this family of materials could contain excellent candidates for piezo- and ferroelectric applications.

## Abstract 2

Metal thiocyanate hybrid perovskites can have multiple simultaneous complex orderings and combining these orderings appropriately can produce non-centrosymmetric structures.

## Graphical abstract

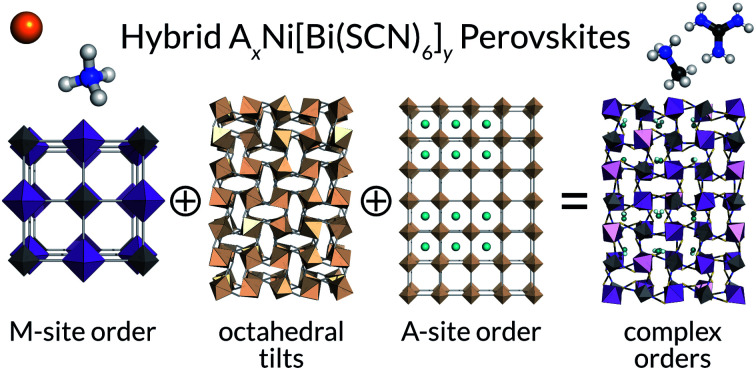

## 1 Introduction

Molecular perovskites, perovskites of composition AMX_3_ where at least one of A, M or X is molecular, have additional degrees of freedom which can produce orderings impossible in atomic perovskites.^1^ These new orderings provide novel routes for materials to respond to external stimuli. One area of particular interest is using the molecular components to create electrical polarisation, without the need for the second-order Jahn–Teller distortions or stereoactive lone pairs that drive piezo- and ferroelectricity in atomic perovskites, *e.g.* BaTiO_3_ or Pb(Zr, Ti)O_3_.^2^ Molecular perovskites now possess polarisations and transition temperatures which approach those of inorganic perovskites.^3^ However, their polarity is typically produced by the orientational order of polar A-site cations.^4^ The functionality often bestowed by the MX_3_ framework, *e.g.* ferromagnetism or ferroelasticity, therefore usually couples weakly to the polarisation, limiting the scope for multiferroicity.

Generating polarisation *via* collective distortions of MX_3_ framework is difficult as the conventional cooperative tilts of the MX_6_ octahedra are intrinsically non-polar. However, by combining octahedral tilts with other symmetry-breaking orders, such as A-site or M-site occupational order, we can generate polar structures: the so-called hybrid improper ferroelectrics.^5^ Furthermore, recent work has shown that the unusual framework distortions possible in molecular perovskites, such as unconventional tilts and columnar shifts, while non-polar, offer new routes to polarity.^1^ Creating materials capable of sustaining new ordering types and sustaining multiple simultaneous orders is therefore a powerful method for generating novel function.^6^

Gaining control over these orders, both individually and separately, remains one of the challenges of solid-state chemistry. One key guiding parameter is the tolerance factor 
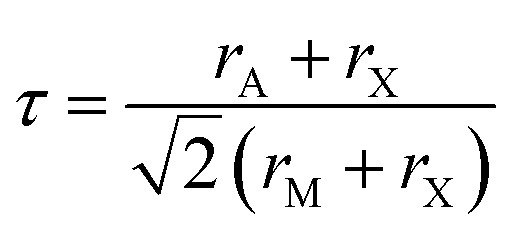
 where *r*_A_ is the radius of the A cation, *r*_M_ is the radius of the M cation and *r*_X_ is the radius of the X anion, which quantifies our intuition that the A-site cation has to fit well into the MX_3_ cage. It indicates whether AMX_3_ is likely to be a perovskite, rather than (*τ* > 1) 1D or 2D structure types (*e.g.* hexagonal ‘perovskite’) or (*τ* < 0.8) other, dense non-perovskite structure types (*e.g.* ilmenite). Although originally developed for atomic perovskites, the tolerance factor approach can rationalise the structures of a wide-range of molecular perovskites, including formates and alkylammonium metal halides,^8,9^ and its fundamental geometric insight has been generalised to other systems.^10,11^*τ* is also linked to the size of the octahedral tilts, as smaller *τ* tends to require large tilts to retain a dense structure. However, creating new function requires controlling the relative sense of the tilts, *i.e.* whether each layer of octahedral tilts rotates in same sense as the next (*a*^+^ in the Glazer notation^12^), or opposite sense (*a*^−^), not just their magnitude. This remains challenging to predict for new perovskites.^13^

*τ* can be readily tuned by creating solid solutions of cations (or cations and vacancies) on the A or M site, as the entropy of mixing stabilises these phases at the high synthesis temperatures used. Conversely, this means that cation-ordering is uncommon, particularly on the A-site.^14^ A-site order is most often stabilised by large size differences between A-site cations, especially the extremal size difference between a vacancy and a cation, and therefore typically produces layered order which minimises the local strain, *e.g.*
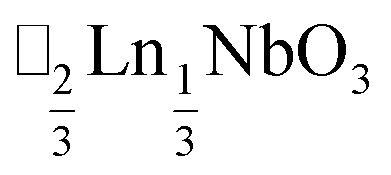
,^15^
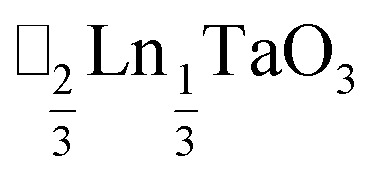
 (ref. 16) and 
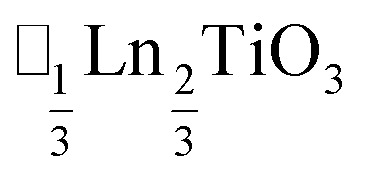
 (ref. 17) (□ = vacancy, Ln = lanthanide). M-site order is stabilised by large charge differences, which favours rocksalt order (the ‘double perovskite’ structure) for electrostatic reasons.^14^ Simultaneous control of these A-site and M-site occupational orders to make so called ‘double double’ perovskites requires therefore specific chemical compositions, but can produce new function *e.g.* polarity in NaLaMnWO_6_.^18^

Rarer A-site occupational orders are typically stabilised by coupling the A-site order to octahedral tilt distortions of the MX_3_ framework. Notably, CaFeTi_2_O_6_ has the unusual *a*^+^*a*^+^*c*^−^ tilt sequence which facilitates columnar A-site order^19^ and the *a*^+^*a*^+^*a*^+^ tilt sequence found in CaCu_3_Ti_4_O_12_ stabilises 3 : 1 Cu_3_Au-type A-site order.^20^ These challenges mean the synthesis of double double perovskites often requires specialist conditions such as high pressure.^14,21^

Molecular perovskites are fertile ground for the exploration of multiple simultaneous orders because of their chemical diversity, low temperature syntheses, and the toolbox of crystal engineering (*e.g.* H-bonding).^22^ We focus in this paper on the family of perovskite-like materials derived from thiocyanate, A_*x*_{M[M′(SCN)_6_]}, of particular interest for their catalytic and optical function.^7,23–26^ These NCS-perovskites have complete M-site order, due to the difference between N- and S-termini of the ligand, and have large tilts due to the frontier molecular orbitals of the NCS^−^ ligand.^7^ The robustness of these distortions means NCS-perovskites are an ideal platform for exploring complex orderings.

**Fig. 1:**
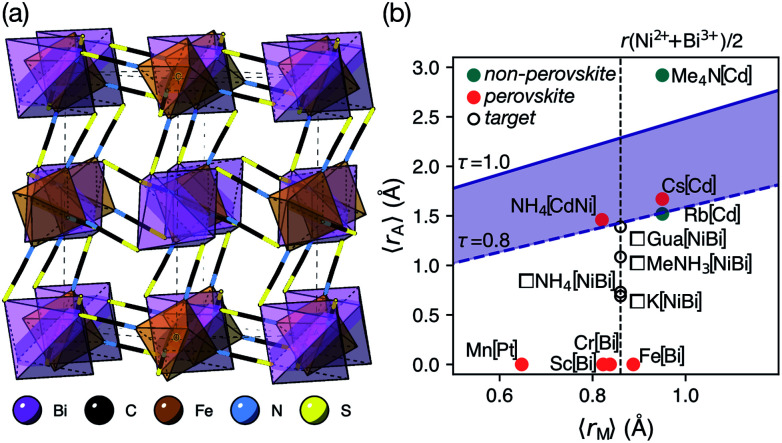
(a) Crystal structure of Fe[Bi(SCN)_6_] viewed along the [110] direction.^7^ (b) Extended tolerance factor plot for A_*x*_M(NCS)_3_ structures including known and target phases, with the M site cations shown in square brackets. The lower limit of *τ* = 0.8 is found not to hold for NCS-perovskites [ESI Section 3‡].

Like the related cyanide Prussian blue analogues, NCS-perovskites are stable in the ‘empty perovskite’ ReO_3_ structure.^27^ Indeed, there are only two reported NCS-perovskites containing A-site cations: Cs{Cd(NCS)_3_} (ref. 24) and the double perovskite (NH_4_)_2_{Ni[Cd(SCN)_6_]}.^23^ Calculation of *τ*, using empirical cation sizes for molecular ions,^8^ suggests that organic A-site cations will likely be too large for A{M(NCS)_3_} perovskites [Fig. 1 and ESI Section 3‡]. Indeed, for the well studied A^+^{Cd(NCS)_3_} composition, although changing A results in a wide variety of structures, including ferroelastics and non-linear optical materials,^28–32^ only Cs{Cd(NCS_3_)} adopts the perovskite structure. Introducing A-site vacancies allows us to reduce *τ* and thereby stabilise NCS-perovskites containing larger organic cations, increasing the range of available orderings.

In this paper, we report the synthesis and structure-determination of a series of A-site vacancy-ordered double perovskites 
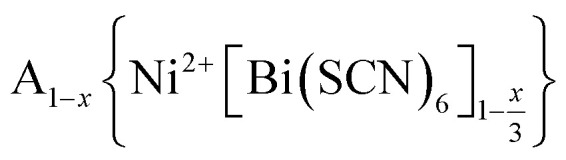
, A = K^+^, NH_4_^+^, MeNH_3_^+^ and C(NH_2_)_3_^+^ (Gua^+^). We thus show that larger A-site cations can be incorporated into NCS-perovskites. We demonstrate that the identity of A-site cation plays a critical role in the structure of thiocyanate perovskites, and that new and unusual combinations of A-site order, M-site order and octahedral tilt patterns can be readily achieved in these materials. In particular, we show using a combination of X-ray and neutron diffraction and density functional theory (DFT) calculations that the A-site cation order and octahedral tilts are strongly coupled. Inspired by these structures, we use symmetry analysis and DFT calculations to suggest the combination of complex orders found in thiocyanate perovskites could be used to produce cooperative properties such as piezoelectricity.

## 2 Results

### 2.1 (NH_4_){Ni[Bi(SCN)_6_]}, 1, and K{Ni[Bi(SCN)_6_]}, 2

**Fig. 2:**
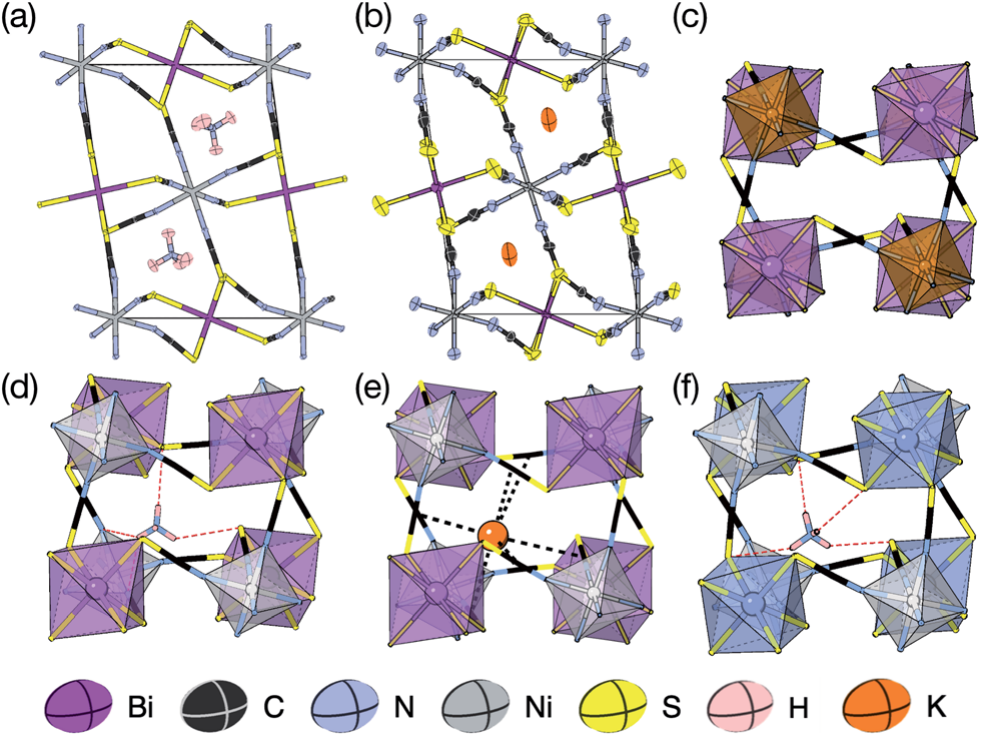
(a) Neutron single crystal structure of 1 at 20 K and (b) X-ray single crystal structure of 2 at 180 K. Anisotropic atomic displacement factors shown as ellipsoids. (c–f) Single pseudocubic cages and guest (where present) for (c) Fe[Bi(SCN)_6_],^7^ (d) 1 NH_4_{Ni[Bi(SCN)_6_]}, (e) 2 K{Ni[Bi(SCN)_6_]} and (f) (NH_4_)_2_{Ni[Cd(SCN)_6_]}.^23^ H-bonds are indicated by dashed red lines, and close contacts by dashed black lines.

We were able to grow large single crystals of phase 1 and 2 by slow evaporation of butanone solutions of the desired stoichiometry. Solution of the structure using single crystal X-ray diffraction (SCXD) showed that the phases are isostructural, as is often found for K^+^ and NH_4_^+^ compounds [Fig. 2]. They consist of octahedrally coordinated NiN_6_ and BiS_6_ cation polyhedra connected by NCS^−^ into a 3D Ni[Bi(SCN)_6_]^−^ framework with ReO_3_ topology, just as in the parent M[Bi(SCN)_6_], M = Sc, Cr, Fe phases. K^+^/NH_4_^+^ cations are present in half of the pseudocubic Ni_4_Bi_4_(NCS)_12_ cages.

This structure is a 
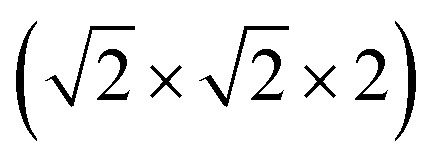
 supercell of the hypothetical primitive cubic *Pm*3̄*m* aristotype AM(NCS)_3_, and its structure derives from this *Pm*3̄*m* structure (using the setting with the A-site at the origin) through four symmetry-lowering distortions. The M-site cations have rocksalt order and this order transforms as the R_2_^−^ irreducible representation (irrep).^33,34^ The A-site cations have columnar order (transforming as the M_1_^+^ irrep), and there is no evidence of site-mixing from single-crystal diffraction. The presence of simultaneous A- and M-site occupational order means that 1 and 2 are double double vacancy-ordered perovskites, □A{Ni[Bi(SCN)_6_]}. 1 and 2 possess the common *a*^−^*a*^−^*c*^+^ (Glazer) or GdFeO_3_ octahedral tilt sequence (which transforms as a combination of the R_5_^−^ ⊕ M_2_^+^ irreps),^12^ which is the same as the parent M[Bi(SCN)_6_] phases and the related M-site defect-ordered NCS-frameworks 
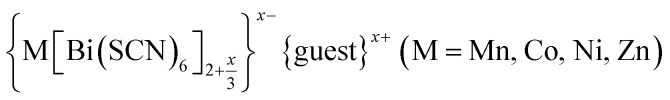
.^7,25^ The combination of octahedral tilting and rocksalt M-site order leaves all pseudocubic Ni_4_Bi_4_(SCN)_12_ cages still equivalent by symmetry, meaning that the A-site cation ordering may not be viewed being drive by these three distortions alone. The A-site cation ordering therefore lowers the space-group symmetry further, from *P*2_1_/*n* to *P*1̄, and in addition produces a large shear strain compared to the M[Bi(SCN)_6_] structures (*α* ≈ 97° *vs. α* = 90°).

SCXD refinement allowed us to tentatively locate the positions of the H atoms and demonstrate that the orientation of the NH_4_^+^ cation in 1 is ordered. Single crystal neutron diffraction (SCND) measurements on a large single crystal (16 mm^3^) at 20 K, carried out using instrument D19 at the ILL, allowed accurate determination of the H atom positions and its anisotropic atomic displacement parameters, which were consistent with those observed *via* SCXD. Variable temperature unit cell measurements between 20 K and 260 K and an additional full collection at 260 K found no evidence of any structural phase transitions in this range. Refinement of the 260 K dataset confirmed the presence of NH_4_^+^ orientational order throughout this temperature range. The ordering of the NH_4_^+^ cation does not lower the symmetry of 1 beyond the symmetry of compound 2.

We further investigated the energetic driving force for the observed A-site order using DFT calculations of K{Ni[Bi(SCN)_6_]}. We carried out geometry optimisations of supercells containing the seven simplest A-site cation orders: rocksalt, layered (with layer normals along the *a*, *b* and *c* directions) and columnar (with columns running along the *a*, *b* and *c* directions), generated from supercells of the Fe[Bi(SCN)_6_] structure [ESI Section 4‡]. The lowest energy structure was the observed columnar [001] order [Table 1], which also had significantly more anisotropic strain than all other orderings [ESI Table 4‡]. The stability of each cation order thus depends on the how easily the parent framework can deform to accommodate a given order.

**Table 1:** DFT-derived energy per formula unit for different K^+^ orderings in K{Ni[Bi(SCN)_6_]}

A-site order	Δ*E* (kJ mol^−1^ per f.u.)
Rocksalt	5.4
Columnar [001]	1.2
Columnar [010]	12.8
Columnar [100]	12.8
Layered (001)	9.4
Layered (010)	8.6
Layered (100)	8.7
Expt.	0.0

### 2.2 (MeNH_3_){Ni[Bi(SCN)_6_]}, 3

**Fig. 3:**
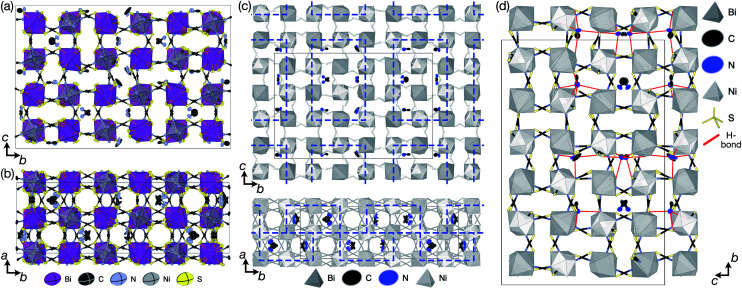
X-ray single crystal structure of 3 at 120 K viewed along the (a) *a* and (b) *c* directions. (c) Block A-site cation order in 3 highlighted by blue rectangles. (d) Guest–framework H-bonding in 3 highlighted by red lines.

We obtained single crystals of MeNH_3_{Ni[Bi(SCN)_6_]}, 3, using a route analogous to that used for 1 and 2. SCXD studies of MeNH_3_{Ni[Bi(SCN)_6_]} revealed that it also crystallises as a double double vacancy-ordered perovskite which, like 1 and 2, has a structure derived from an ReO_3_-type parent by introducing cations into half of the pseudocubic cages [Fig. 3]. However, 3 has a more complex structure than 1 and 2 and its unit cell is a (2 × 6 × 4) monoclinic *P*2/*n* supercell of the *Pm*3̄*m* aristotype (*i.e.* is 12 times larger than the structures 1 and 2), due to an unusual ordering of the MeNH_3_^+^ cations and complex octahedral tilting. The complexity of the order, together with the high metric pseudosymmetry 
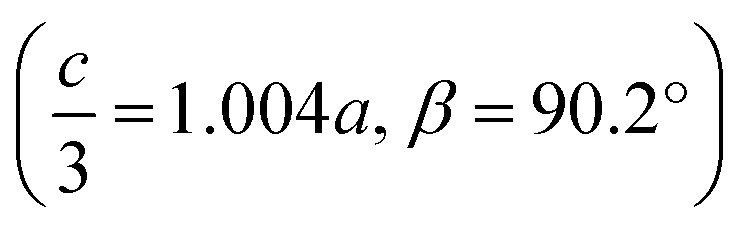
, means that 3 has a high propensity for twinning. We found that twinning is common around both the [001] and [110] lattice directions, which made structure solution and refinement challenging. Using SCXD data collected on a small crystal (65 × 55 × 22 μm) which only had a minor twin component (<20%, 180° twin around the [001] direction), we were able to produce a stable refinement for the structure.

We were not able to locate the hydrogen atoms on the MeNH_3_^+^ cation and our assignment of the polarity of MeNH_3_^+^ cation, *i.e.* which atom was carbon and which nitrogen, was thus tentative. We therefore carried out a series of SCND studies on large (≈1 mm^3^) single crystals using instrument D19 at the ILL. These measurements did not allow us to definitively answer these questions because we were unable to obtain an untwinned crystal of sufficient size, but did confirm both the space group symmetry and broad structural features observed *via* SCXD.

**Fig. 4:**
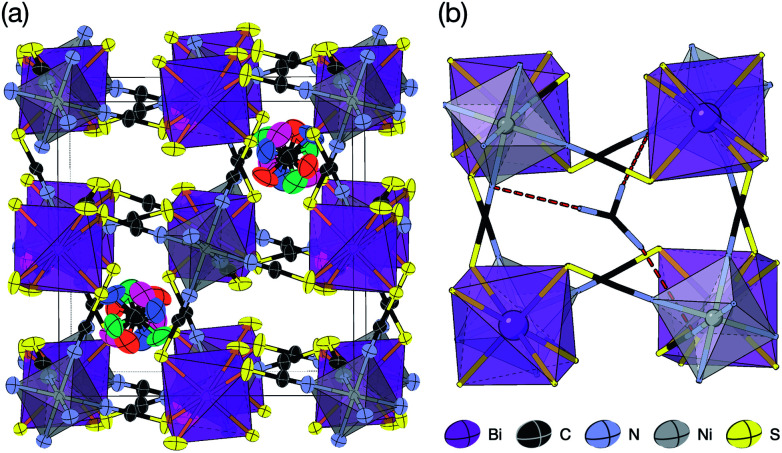
Synchrotron X-ray single crystal structure of 4 at 100 K. (a) Full structure with atomic displacement parameters and metal polyhedra shown. Each Gua^+^ orientation is shown in a different colour. (b) A single pseudocubic cage with one of the four Gua^+^ orientations and H-bonding to the framework is indicated by dashed red lines.

Synchrotron X-ray diffraction data measured on a room temperature polycrystalline powder sample of 3 carried out at beamline I11 at Diamond Light Source could be indexed completely by the *P*2/*n* (2 × 6 × 4) supercell, with no peaks unaccounted for. Rietveld refinement using the model derived from SCXD data gave quantitative agreement [ESI Section 2.3;‡Fig. 3 and 4]. Notably, excluding the MeNH_3_^+^ cations from the model significantly degraded the quality of fit (*R*_wp_ increased from 2.97 to 3.59).

The MeNH_3_^+^ cations are present in blocks of (1 × 2 × 3) pseudocubic cages surrounded by cages containing vacancies [Fig. 3(c)], with complete occupational order. Half of the MeNH_3_^+^ had disordered orientations, however, with one third disordered about high symmetry positions and one sixth showing disorder unrelated to the crystal symmetry. Additionally, eight symmetry independent NCS^−^ ions were disordered over two sites. The atomic displacement parameters for the MeNH_3_^+^ cations were significantly larger than that for the framework atoms, indicative of either dynamic or static disorder.

We therefore carried out DFT geometry optimisations to understand the energy scales of the disorder in this system. We created an ordered model of the structure with *P*1 symmetry derived from our diffraction model and geometry optimised it to confirm its stability. Next, we systematically swapped the carbon and nitrogen atoms of each the eight symmetry independent MeNH_3_^+^ cations, one cation at a time, and geometry optimised each of the eight resultant structures. Our diffraction-derived model had the lowest energy of the nine configurations explored. The energy penalty for flipping the MeNH_3_^+^ varied significantly, from Δ*E*_CN_ = 10.4 kJ mol^−1^ up to Δ*E*_CN_ = 22.7 kJ mol^−1^ [ESI Table 5‡]. The size of the energy penalty correlated with the degree of crystallographic disorder: the three well-ordered cations (MA-3, MA-5 & MA-8, numbering corresponding to the CIF file) had three highest Δ*E*_CN_, averaging Δ*E*_CN_ = 19.5 kJ mol^−1^, whereas the five disordered cations averaged Δ*E*_CN_ = 12.9 kJ mol^−1^. The energetic driving force for A-site vacancy/cation order was an order of magnitude larger, with the energy for displacing an MeNH_3_^+^ cation to an adjacent cage being Δ*E*_A□_ = 102 kJ mol^−1^. This displacement also perturbed the octahedral tilt pattern, as one thiocyanate ligand was moved out of the cage to accommodate the MeNH_3_^+^ cation.

We used ISODISTORT^35^ to carry out symmetry mode analysis of 3. We first investigated the Ni[Bi(SCN)_6_]^−^ framework and found that the distortion of the structure from the hypothetical parent *Pm*3̄*m* structure (from the rocksalt M-site ordered *Fm*3̄*m* structure) could be described well by six symmetry-adapted distortion modes in addition to rocksalt M-site order, one of which describes the global contraction of the structure Γ_1_^+^ (Γ_1_^+^), and four which describe cooperative rigid octahedral rotations: M_2_^+^ (X_3_^+^), 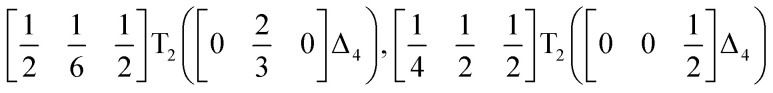 and R_5_^−^ (Γ_4_^+^) [Table 2].

**Table 2:** Octahedral distortion modes in MeNH_3_{Ni[Bi(SCN)_6_]}^12,34,36^

Irrep.^34^ from *Pm*3̄*m* (*Fm*3̄*m*)	*k*	Glazer tilt^12^	Peel tilt^36^
M_2_^+^ (X_3_^+^)	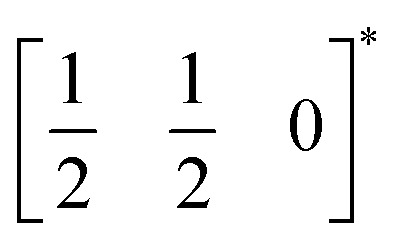	*a* ^+^00	C
T_2_ (Δ_4_)	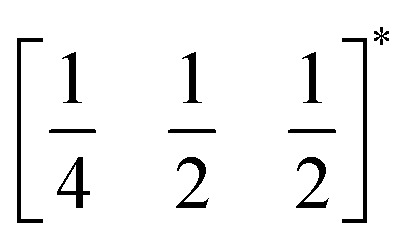	0*b*^++−++−^0	CCCAAA
T_2_ (Δ_4_)	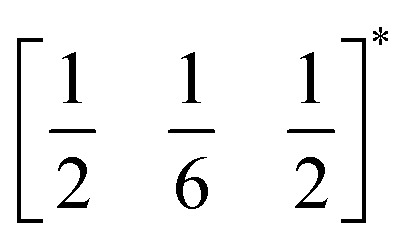	00*c*^+−+−^	CCAA
R_5_^−^ (Γ_4_^+^)	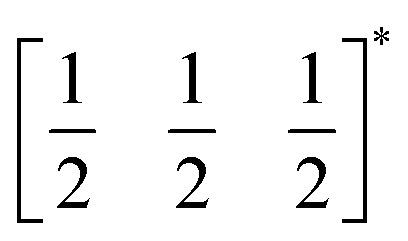	0*b*^−^0	CA

The two T_2_ modes are notable as they are not zone corner Brillouin modes, and correspond to complex, but conventional, octahedral tilts. All conventional tilting modes will produce a doubling of the unit cell in the tilt plane (as the rotations of adjacent octahedra within the plane have opposite senses), but adjacent layers need not tilt with the same sense. The two highest symmetry octahedral tilting modes are: all layers being in phase, *a*^+^ in the Glazer tilt notation^12^ and [C] in the notation of Peel *et al.*^36^ which transforms as a M_2_^+^ distortion mode, and each layer alternating in its sense rotation, a^−^, [CA] and R_5_^−^. In 3, the tilts normal to the *b* and *c* axes repeat after six and four layers (respectively) and are therefore complex. In total, the tilt sequence for this perovskite is [C][CCCAAA][CCAA] (Peel), or *a*^+^*b*^++−++−^*c*^+−+−^ (extended Glazer), where both notations are shown for clarity.

These complex tilts observed along the *b* and *c* directions are a form of nanoscale ‘tilt-twinning’: sequences of the same tilt sequence interrupted by a tilt of the opposite kind. Symmetry analysis showed that the combined presence of the M_2_^+^, 
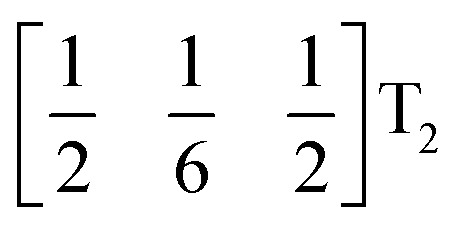
 and 
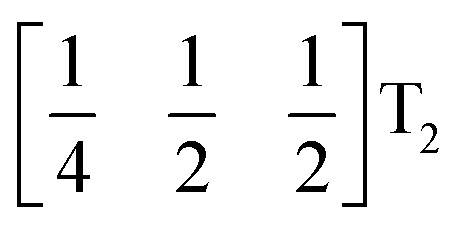
 tilting modes together with rocksalt M-site order (R_2_^−^) was sufficient to produce the observed *P*2/*n* (2 × 6 × 4) structure. These modes are therefore likely the primary order parameters, with the R_5_^−^ (Γ_4_^+^) mode being a secondary order parameter. The A-site order can only be described by secondary order parameters arising from all three tilts, with any pairwise combination being insufficient, which suggests that it is the final distribution of anions ordering produced by the complete octahedral tilt pattern which is responsible for the observed ordering.

### 2.3 □_9_Gua_3_{Ni_6_[Bi(SCN)_6_]_5_}, 4

Slow evaporation of a butanone solution containing Gua(SCN), Ni^2+^ and Bi(SCN)_6_^3−^ in a 6 : 1 : 1 ratio yielded large single crystals of compound 4. We were again able to determine its structure using SCXD, which revealed that it was also a vacancy ordered double perovskite, in space group *Pn*3̄, a (2 × 2 × 2) supercell of the *Pm*3̄*m* aristotype [Fig. 4]. However, 4 contains only half the number Gua^+^ cations anticipated and refinement of the occupancies showed that this structure contained a significant fraction of M-site vacancies 
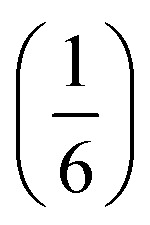
, refined formula Gua_0.5_ Ni[Bi_0.900(4)_(S_0.860(6))_N_0.841(12)_N)_6_], corresponding to □_9_Gua_3_Ni_6_[Bi(SCN)_6_]_5_. This reduced A-site occupancy and the presence of vacancies also accords with the lower volume of compound 4 compared to 3, despite 4 containing a significantly larger A-site cation [Table 3].

**Table 3:** Pseudocubic averaged lattice parameters and unit cell volume in the *Pm*3̄*m* aristotype (*i.e.* the volume of one pseudocubic cage) for compounds 1–4 and the empty A-site Fe[Bi(SCN)_6_] analogue, demonstrating how unit cell per volume changes with A site cation

A-site	*a*_*Pm*3̄*m*_ (Å)	*V*_*Pm*3̄*m*_ (Å^3^)	*T* (K)
□ (Fe[Bi(SCN)_6_])	5.9865(2)	214.518(5)	180
K^+^	6.001(2)	216.12(2)	180
NH_4_^+^	6.011(2)	217.24(2)	120
MeNH_3_^+^	6.1263(3)	229.935(19)	120
Gua^+^	6.06264(3)	222.8361(13)	100

The observed space group of *Pn*3̄ is that expected for the *a*^+^*a*^+^*a*^+^ tilt sequence,^37^ and indeed analysis using ISODISTORT confirmed this tilt sequence is adopted by compound 4. This tilt sequence is well known for other perovskites with 1 : 3 A-site cation ratio.^14^ In addition each Gua^+^ cation is disordered over four positions. Our single crystal diffraction measurements are consistent with both static and dynamic disorder, but the absence of any A-site order at 120 K, well-below typical ordering temperatures for Gua^+^ containing molecular perovskites,^38,39^ suggests that this disorder is static.

Synchrotron single crystal X-ray diffraction measurements showed the presence of weak structured diffuse scattering, consisting of rods lying along 〈100〉^*^ type directions [ESI Fig. 2‡]. The intensity of the diffuse scattering decayed with increasing scattering vector, *Q*, implying that the diffuse scattering is produced primarily by correlated substitutional disorder, most likely vacancy ordering, rather than displacive disorder. The asymmetric distribution of intensity around each Bragg peak additionally suggests that the structure relaxes around these vacancies.^40^ Future analysis will focus on gaining quantitative understanding of vacancy order.

### 2.4 Gua(SCN) and hydrogen bonding

**Fig. 5:**
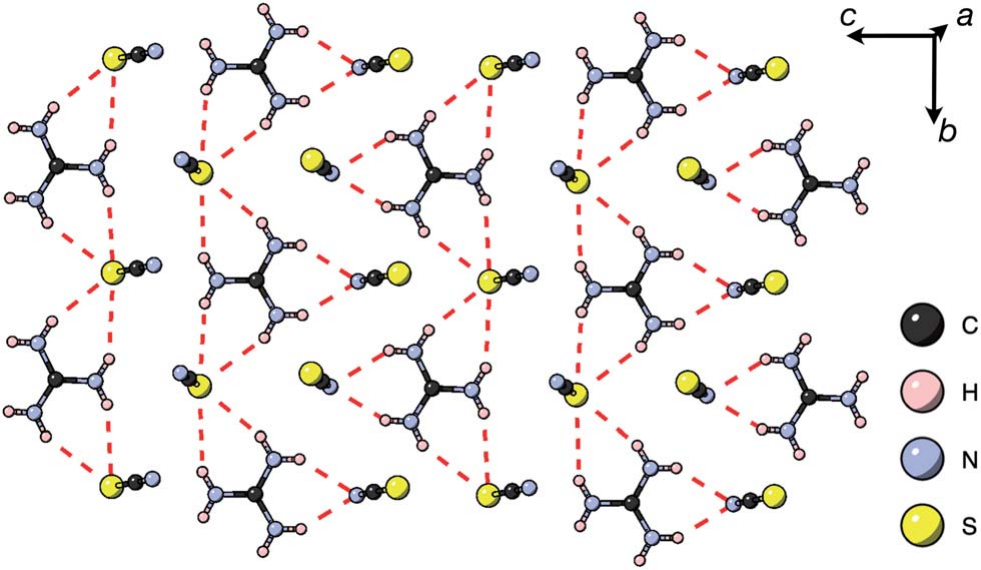
A H-bonded layer of Gua(SCN) viewed along the a* direction. H-bonds indicated by dashed red lines.

Thiocyanate is a hydrogen bond (H-bond) acceptor, but there are comparatively few studies of its hydrogen bonding propensity.^41^ To benchmark the hydrogen bonding between the A-site cations and the NCS^−^ in these materials we therefore examined a hydrogen bond rich material, Gua(SCN).^42^ We redetermined the structure using a crystal present in commercially supplied GuaSCN. Gua(SCN) crystallises in space group *P*1̄, with *Z*′ = 2. Its structure arises largely from the need to optimise its H-bonding, as it comprises H-bonded layers in the *bc* plane slip-stacked along the *a* direction [Fig. 5]. These layers consist of a honeycomb lattice of NCS^−^ ions, which lie approximately normal to the layer, and a bilayer of Gua^+^ cations positioned at the centre of the honeycomb voids and which form a triangular lattice. Half of the NCS^−^ ions point up and half down, and this up-down pattern is stripe-ordered along the b* direction.

Each Gua^+^ cation forms charge-assisted bifurcated hydrogen bonds to three NCS^−^ ions: one to an N-terminus and two to an S-terminus. Likewise, the NCS^−^ forms hydrogen bonds to three Gua^+^ cations, one through its N-terminus and two through its S-terminus. These hydrogen bonds also cause the NCS^−^ ions to tilt away from the a* direction along the *c* direction towards the Gua^+^ cations. The average distances from H-bond donor hydrogen to acceptor atom are *d*_H⋯N_ = 2.30(5) Å and *d*_H⋯S_ = 2.71(8) Å (standard deviation in parentheses); and the average distances from H-bond donor nitrogen to acceptor atom are *d*_NH⋯N_ = 3.07(4) Å and *d*_NH⋯S_ = 3.50(6) Å. These are consistent with previous investigations of H-bonding in thiocyanate compounds.^41^

These distances, together with a search of short contacts present in the Cambridge Structural Database, guided our investigation into the presence of H-bonding in 1, 3 and 4. We searched for all close contacts from the donor nitrogen to NCS (*d*_NH⋯N_ < 3.2 Å, *d*_NH⋯S_ < 3.6 Å), as donor hydrogen atoms were only accurately located in 1. We found that strong hydrogen bonds are present for each compound, and are likely to be structure-directing.

There are a number of close contacts between the NH_4_^+^ cation and NCS^−^ anions in compound 1, corresponding to a bifurcated NH⋯N⋯HN H-bond and a NH⋯S H-bond [Fig. 2(d)]. 3 contains NH⋯N close contacts between five symmetry independent MeNH_3_^+^ cations and NCS^−^ ligands (MA-1, MA-3, MA-5, MA-7 & MA-8, numbering as in CIF file) and NH⋯S close contact between six MeNH_3_^+^ cations and NCS^−^ (MA-1, MA-3, MA-4, MA-5, MA-7, MA-8) [Fig. 3(c)]. These include the three crystallographically well ordered MeNH_3_^+^ cations (MA1, MA3, MA8) suggesting H-bonding plays a key role in holding the A-site cations in place. In compound 4 each Gua^+^ N atom donates 1 H-bond to an NCS^−^ N acceptor (*d*_NH⋯N_ = 3.101 Å) [Fig. 4(b)]. As each cage contains four distinct orientations of the Gua^+^ cation, and is surrounded by twelve NCS^−^ ligands, this means one quarter of all NCS^−^ will be H-bond acceptors.

## 3 Discussion

### 3.1 Orientational order of A-site cations

Compounds 3 and 4 are the first NCS-perovskites containing organic A-site cations, adding to the existing NCS-perovskites containing inorganic A-site cations, Cs{Cd(NCS)_3_}^24^ and (NH_4_)_2_{Ni[Cd(SCN)_6_]},^23^ and those containing metal coordination-complexes on vacancy sites, Mn_2_Bi(SCN)_7_·7H_2_O and Co_9_Bi_6_(SCN)_36_(H_2_O)_38_.^25^ Perovskites containing organic A-sites cations are of particular interest as these organic cations can possess intrinsic electric dipoles (*e.g.* MeNH_3_^+^) and quadrupoles (*e.g.* Gua^+^). The orientational order of organic A-site cations can thus generate electrical polarisation, either directly through ferrodipolar order, as in the formate perovskites,^4^ or indirectly through coupling of ferroquadrupolar order to other order parameters, for example (Gua){Cu(HCO_2_)_3_}.^1,38^

We did not find polar orientational order in these new perovskites, and complete orientational order was only present in 1, as 3 shows partial disorder and 4 complete disorder. Our variable temperature diffraction studies found no evidence of any phase transitions below 260 K, implying that the observed A-site disorder is static, which for compound 4 is likely related to the presence of M-site vacancies. Our DFT calculations suggest that orientational order in 3 is moderately favourable as Δ*E*_CN,av_ = 16 kJ mol^−1^ (0.17 eV ≈ 6 kT at room temperature). Careful structural examination revealed that hydrogen bonding is an important factor in the structures of these materials, as in other molecular perovskites,^43^ and indeed, Δ*E*_CN,av_ is comparable to the H-bonding energies found in formate perovskites.^22^ This suggests that temperature-induced phase transitions might be uncovered with careful comprehensive variable temperature structural and calorimetric studies, as in (NH_4_)_2_{Ni[Cd(SCN)_6_]}, which undergoes an order–disorder transition associated with the NH_4_^+^ cation at around 120 K.^23^ Optimisation of the orientational order of A-site cations towards ferroic order might be possible through crystal-engineering, by tuning the hydrogen-bonding or introducing halogen-bonding moieties,^44^ and by deepening our understanding of the role of framework entropy in NCS-perovskites.^45^

### 3.2 Coupling between A-site occupational order and octahedral tilting

In atomic perovskites, the tolerance factor has been successfully used not only to suggest whether a composition will be a perovskite, but also to provide a first indication of the magnitude of octahedral tilts, *e.g.* CaTiO_3_ adopts the distorted *a*^−^*a*^−^*c*^+^ tilt system at room temperature, whereas SrTiO_3_ is cubic with no tilts. Although the tolerance factor approach explains which molecular AMX_3_ frameworks are likely to crystallise with a perovskite structure,^8,9^ it does not account for either the magnitudes or kinds of framework distortions observed.^46,47^ For example, in the series of formate perovskites A{Mn(HCO_2_)_3_} where A^+^ = Rb^+^,^48^ CH_3_NH_3_,^49^ (CH_3_)_2_NH_2_ (ref. 50) and (CH_2_)_3_NH^51^ (arranged in increasing size of A^+^/increasing *τ*), there is no systematic trend in the size or pattern of the octahedral tilting, respectively: *a*^−^*a*^−^*c*^−^, *a*^−^*a*^−^*c*^+^, *a*^−^*a*^−^*c*^−^ and *a*^−^*a*^−^*c*^+^.

The scarcity of NCS-perovskites has thus far prevented investigation of the relationship between cation size and tilts. We find, contrary to simple geometric arguments, that the average size of the NiN_6_ octahedral tilt (measured by the ∠N–Ni–Bi angle) and the BiS_6_ tilt (∠S–Bi–Ni) change very little for these four compounds from the parent M[Bi(SCN)_6_] frameworks. This conforms to the general finding that the metal-thiocyanate bond-angles do not vary in NCS-perovskites and that guest–framework interactions exert only second-order effects.^7,23,25,26^ Compound 4 crystallises with both A- and M-site vacancies, suggesting that there is a maximum average size of A-site cation that can be incorporated within the {Ni[Bi(SCN)_6_]}^−^ framework and providing further evidence of the ease of formation of [Bi(SCN)_6_]^3−^ vacancies in these materials. The tolerance factor therefore may provide a useful upper bound on cation size for NCS-perovskites (the lower bound not being meaningful due to the variety of ReO_3_ structure NCS-frameworks), but we have not found it to be predictive of the tilts or A-site ordering—just as for other molecular perovskites.

### 3.3 A-site vacancy order

A-site vacancy ordered molecular perovskites are rare, as the additional structural degrees of freedom often mean other structure types are favoured for high vacancy concentrations: for example AM^II^M^III^(HCO_2_)_6_ compounds adopt niccolite-type structures.^52^ The Prussian blue analogue cyanides can accommodate the complete range of A-site compositions, which has been exploited for their potential as battery electrode materials,^53^ but the A-sites are typically disordered. Some degree of rocksalt A-site cation order has been observed in a number of frameworks of approximate composition AM^II^M^III^(CN)_6_ (ref. 54) but this is typically incomplete, perhaps due to the high symmetry of these phases.^55,56^ Perhaps the best described example of A-site order in a vacancy perovskite is the recent report of □_0.5_H_2_DABCO_0.5_{Mn(H_2_PO_2_)_3_}, H_2_DABCO^2+^ = 1,4-diazabicyclo[2.2.2]octane-1,4-diium, where vacancies order into {111}_cubic_ layers presumably, as in A-site vacancy oxides, to minimise strain.^47^

It is therefore noteworthy that A-site vacancy order appears to be the rule in NCS-perovskites, rather than the exception. 1–4 all possess complete A-site order and these orderings are unusual for perovskites molecular or otherwise: in 1 and 2 the cations have columnar order; in 3 the MeNH_3_^+^ order into 3 × 2 × 1 blocks and in 4 the cations are present in one quarter of the cages with Cu_3_Au order. The block-order of cations in MeNH_3_{Ni[Bi(SCN)_6_]} is to the best of our knowledge unknown in any other perovskite. It can be related to the nanochequerboard/nanochessboard phases observed in compositionally complex analogues of the rare-earth vacancy perovskites, such as 
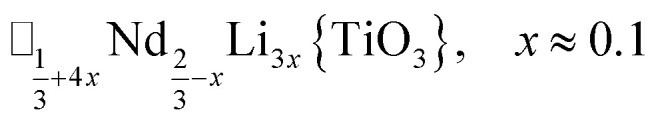
 (ref. 57) and 
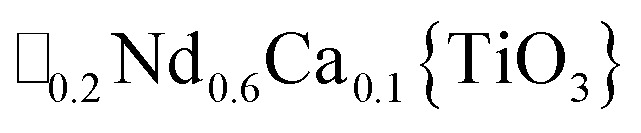
.^58^ These phases have a modulation in the occupancy of the A-site on a *ca.* 5 nm lengthscale. In addition the combination of M-site rocksalt and A-site columnar order found in 1 and 2 has only been reported previously for the high-pressure oxides MnLnMnSbO_6_, Ln = La, Pr, Nd, Sm,^21^ and CaMM′ReO_6_, M = Mn or (Mn_0.5_Cu_0.5_) and M′ = Mn or Fe.^59^

**Fig. 6:**
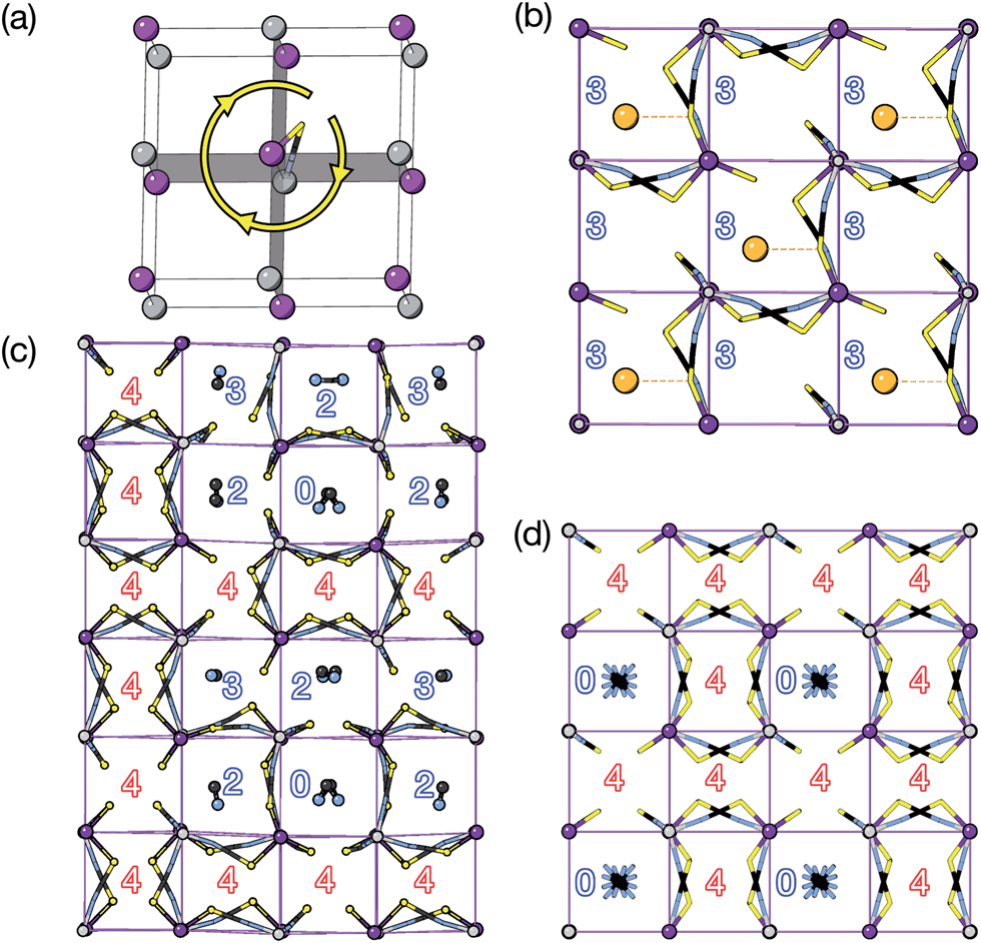
(a) Illustration of how octahedral rotations of the attached cations place a give thiocyanate in one of four pseudocubic cages. (b–d) Single layers of the pseudocubic cages of each structure showing the positions of NCS^−^ ligands and guest cations for compounds 2 (b); 3 (c) and 4 (d). No pseudocubic cage containing four NCS^−^ ligands contains a guest. The numbers indicate how many NCS^−^ ligands lie within each pseudocubic cage. The closest K–S distance is shown for compound 2 by a dashed red line.

We find that for this family of compounds the A-site order and tilts are strongly coupled: each tilt sequence has its own cation order. Columnar order in 1 and 2 accompanies the a^−^a^−^c^+^ tilt, the unique A-site order in 3 is accompanied by the unique complex *a*^+^*b*^++−++−^*c*^+−+−^ tilt, and the Cu_3_Au order occurs with *a*^+^*a*^+^*a*^+^ tilt. One possible reason for this can be seen in the distribution of NCS^−^ anions between pseudocubic cages [Fig. 6]. Each NCS^−^ must lie within one of four adjacent pseudocubic cages, with which cage it lies within determined by the tilting of two metal octahedra it is connected to [Fig. 6(a)]. Each cage is bounded by 12 thiocyanates, so on average a cage contains three thiocyanates. In 4 one quarter of the cages contain no NCS^−^, with 
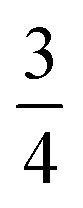
 containing four thiocyanates [Fig. 6(d)]. We find that the pseudocubic cages containing no NCS^−^ are the cages containing Gua^+^ cations, whereas the pseudocubic cages containing four NCS^−^ contain no A-site cations. This correlation likely arise from simple reasons of sterics: there is not enough space in the cages containing four thiocyanates for an A-site cation. This approach is in agreement with previous rationalisations of the structures of CaCu_3_Ti_4_O_12_-type perovskites, which also have *a*^+^*a*^+^*a*^+^ tiles and Cu_3_Au A-site order, where the largest cation (*e.g.* Ca^2+^) sits in the cages containing no O^2−^ anions.^20^

The pseudocubic cages in 3 contain 0, 2, 3 and 4 NCS^−^ anions in the ratio 1 : 3 : 2 : 6; each and every pseudocubic cage which does not contain an A-site cation contains four NCS^−^ anions, and every cage containing fewer than four anions also contains an A-site cation [Fig. 6(c)]. This suggests that the complex tilt pattern derives, in part, from the need to rearrange the NCS^−^ anions to accommodate the larger MeNH_3_^+^ cations in the pseudocubic cages. This ability of octahedral tilts to increase the available volume in some cages, at the expense of others, provides an explanation for why the average, rather than maximum, A-site cation size appears to be the key factor for perovskite stability. In contrast, all the cages in 1 and 2 contain three NCS^−^ and so cooperative framework shear therefore is necessary to accommodate the A-site cations. We have applied this anion-in-cage counting method to each of the four simplest 3-tilt patterns (in the approximation that all tilts have equal magnitude) [Table 4]. We find that these tilts, aside from the previously mentioned *a*^+^*a*^+^*a*^+^ tilt sequence, would not be expected to stabilise any particular A-site cation order according to this counting method, as all cages contain three NCS^−^, even if the cages are symmetry distinct. This suggests that complex tilts may be well be favoured in molecular perovskites with large A-site cation size disparities.

**Table 4:** No. of NCS^−^ present in pseudocubic octahedral cages for the four simplest three tilt sequences

Tilt	*n*_cage_ × *n*_NCS^−^_/cage
*a* ^+^ *a* ^+^ *a* ^+^	3 × 4 : 1 × 0
*a* ^+^ *a* ^+^ *a* ^−^	1 × 3
*a* ^+^ *a* ^+^ *a* ^−^	1 × 3 : 1 × 3
*a* ^−^ *a* ^−^ *a* ^−^	1 × 3

### 3.4 Complex tilts

**Fig. 7:**
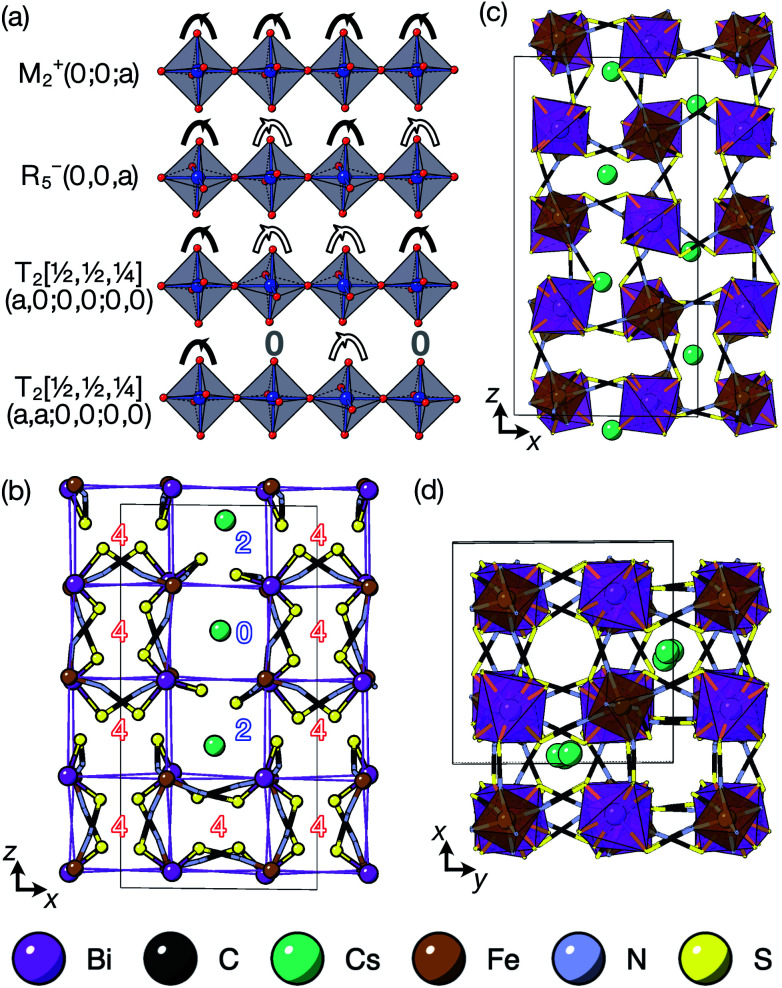
(a) The single-axis octahedral tilts with symmetry labels. (b) A single layer of pseudocubic cages for the piezoelectric *P*4̄2*c* structure, with ligand counts. DFT optimised structure of Cs_3_{Fe[Bi(SCN)_6_]_4_} viewed along the (c) *b* and (d) *c* axes.

The tilts along the *b* and *c* directions in 3 are complex, that is, the repeat distance for the tilt pattern is greater than two unit cells (*i.e.* the tilts transform as a [00*k*]T_2_ irrep) [Fig. 7(a)]. There are very few examples of perovskites with complex tilts,^60,61^ and perhaps the best known is LiNbO_3_, which forms phases possessing tilts with periodicities of four (S-phase) and six (R-phase) unit cells along a single axis.^36^ Complex tilting has also been observed in molecular perovskites,^46^ with incommensurate tilting discovered in Me_2_NH_2_{Co(HCO_2_)_3_}^62^ and complex tilts along multiple axes in Me_2_NH_2_{Mn(H_2_PO_2_)_3_}.^47^ In both cases the tilts are unconventional as they are formed from shifts and/or out-of-phase tilts (where adjacent octahedra tilt in the same sense). Compound 3 is the first perovskite of any kind, to our knowledge, to show complex conventional tilts along multiple axes.

### 3.5 Breaking centrosymmetry with complex tilts

It is well known that simple conventional cooperative octahedral tilts cannot produce non-centrosymmetric structures.^12^ This is only true, however, for Brillouin zone-corner tilts. We show here that tilt patterns containing complex tilts can generate non-centrosymmetric structures, by carrying out symmetry analysis of the structures derived from the simplest example of a complex tilt, the 
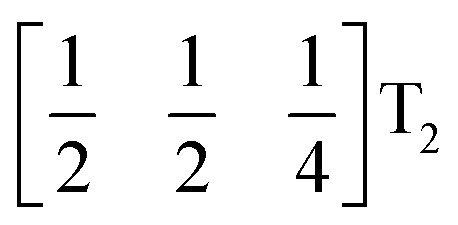
 mode found in the S-phase of LiNbO_3_ (period of for unit cells) [Fig. 7(a)]. We examined the symmetries of the structures produced when a cubic perovskite with M-site rocksalt order (R_2_^−^) is distorted by this 
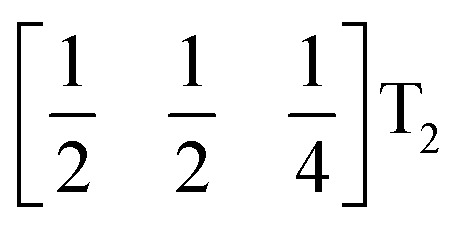
 mode with simple tilts (M_2_^+^ or R_5_^−^) along the other two axes [ESI Fig. 6‡]. The combination of a 
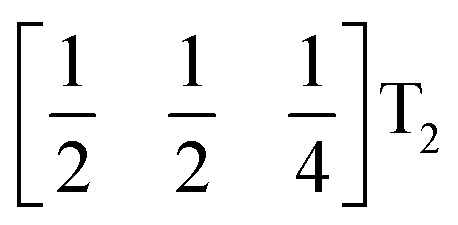
 tilt along the *c* axis, with in-phase M_2_^+^ tilts along the *a* and *b* axes generates a non-centrosymmetric structure with space-group *P*4̄2*c* [ESI Fig. 7‡]. We generated a model of a hypothetical Fe[Bi(SCN)_6_] polymorph possessing these distortions using ISODISTORT and then examined the distribution of NCS^−^ anions. The pseudocubic cages contain 0, 2 or 4 NCS^−^ in the ratio 1 : 2 : 6, suggesting that a structure in which 
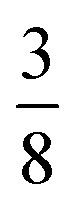
 of the cages were occupied by A-site cations would stabilise this distortion [Fig. 7(b)]. We therefore filled the cages containing 0 or 2 NCS^−^ anions with Cs^+^ cations, producing a model with composition Cs_3_{Fe[Bi(SCN)_6_]_4_} [Fig. 7(c and d)]. Very encouragingly our model, which was constructed only taking into account symmetry analysis and counting cage occupancies, was found to be stable with DFT geometry optimisation. We found that moving a Cs^+^ cation into any of the other cages incurred a significant energetic penalty, suggesting that this cation ordering is energetically preferred. This structure is piezoelectric, with the piezoelectricity arising from the complex tilts along the *c*-axis. The investigation of other stoichiometries and A-site cations will likely be a fruitful route to generating new polar and ferroelectric perovskites.

## 4 Conclusion

In this study we have investigated how the identity of the A^+^ cation determines the coupled A-site occupational order and octahedral tilt distortions of 
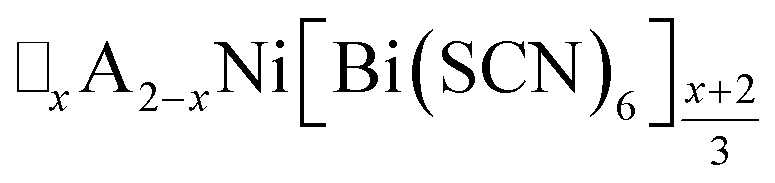
, A = K^+^, NH_4_^+^, MeNH_3_^+^ and Gua^+^, vacancy-ordered perovskites. We have shown that organic cations can be hosted as A-site cations in NCS-perovskites, and that the synthesis of large single crystals of these materials can be achieved *via* facile solution methods. NCS-perovskites have robust M-site order and nearly fixed magnitude conventional octahedral tilts: in this work we have shown that A-site occupational order can also be readily achieved in NCS-perovskites. We demonstrate that, by controlling these three orders at once, we can produce new and unprecedentedly complex perovskite structures, notably the non-Brillouin zone corner tilts and block A-site order found in (MeNH_3_){Ni[Bi(SCN)_6_]}. We have devised a simple counting method for predicting the coupling between octahedral tilts and A-site occupational order, supported by DFT calculations. Finally, we have shown how complex conventional tilts can produce new routes to non-centrosymmetric materials. These results suggest that exploration of NCS-perovskites and complex tilts more generally may uncover further functional behaviour, including ferroelectricity, and anomalous mechanical properties such as negative thermal expansion or negative linear compressibility.

## Author contributions

J. L. and M. J. C. synthesised the samples. M. J. C. analysed the X-ray diffraction data. S. J. A. carried out the synchrotron X-ray diffraction experiments. J. L., M. J. C. and L. C. D. carried out the neutron diffraction experiments. J. L. and L. C. D. analysed the neutron diffraction data. S. L. carried out the DFT calculations. M. S. S. carried out the symmetry analysis. M. J. C. wrote the paper, with input from all other authors.

## Conflicts of interest

There are no conflicts to declare.

## Supplementary Material

SC-012-D0SC06619B-s001

SC-012-D0SC06619B-s002

SC-012-D0SC06619B-s003

SC-012-D0SC06619B-s004

SC-012-D0SC06619B-s005

SC-012-D0SC06619B-s006

SC-012-D0SC06619B-s007

SC-012-D0SC06619B-s008

SC-012-D0SC06619B-s009

SC-012-D0SC06619B-s010
