# Supplementary material for: Controlling multiple orderings in metal thiocyanate molecular perovskites Ax{Ni[Bi(SCN)6]}
Source: Chem Sci. 2021 Jan 15;12(10):3516–25. doi: 10.1039/d0sc06619b (PMC8179531; doi:10.1039/d0sc06619b)
Supplement: SC-012-D0SC06619B-s001 [file SC-012-D0SC06619B-s001.pdf]

Electronic Supplementary Material (ESI) for Chemical Science.
This journal is © The Royal Society of Chemistry 2021

Controlling multiple orderings in metal thiocyanate molecular perovskites A{Ni[Bi(SCN)$_6$]}

Jie Yie Lee[1], Sanliang Ling[2], Stephen P. Argent[1], Mark S. Senn[3], Laura Cañadillas-Delgado[4], and Matthew J. Cliffe [*1]

[1]School of Chemistry, University Park, Nottingham, NG7 2RD, United Kingdom

[2]Advanced Materials Research Group, Faculty of Engineering, University of Nottingham, University Park, Nottingham NG7 2RD, United Kingdom

[3]Department of Chemistry, University of Warwick, Gibbet Hill, Coventry CV4 7AL, United Kingdom

[4]Institut Laue Langevin, 71 avenue des Martyrs - CS 20156, 38042 Grenoble, France.

January 7, 2021

Contents

List of Figures 1

List of Tables 2

1 Synthesis 3
- 1.1 Synthesis of 2 K{Ni[Bi(SCN)$_6$]} . . . 3

2 X-ray Diffraction Measurements 3
- 2.1 Single Crystal X-ray Diffraction . . . 3
- 2.2 Single Crystal Neutron Diffraction . . . 3
- 2.3 Powder X-ray Diffraction . . . 5

3 Tolerance Factor Calculations 10

4 Density Functional Theory Calculations 10

List of Figures

1 Gua(SCN) viewed along b . . . 6
2 Diffuse scattering in **4** with symmetry averaging applied ($m\bar{3}$) . . . 6

[*]matthew.cliffe@nottingham.ac.uk

3 Rietveld fit of synchrotron powder X-ray diffraction data of compound **3**. The calculated pattern is shown in light blue, the experimental data in dark blue and the difference in grey. 7
4 Rietveld fit of synchrotron powder X-ray diffraction data of compound **3**, zoomed in on low Q region. The calculated pattern is shown in light blue, the experimental data in dark blue and the difference in grey. 7
5 Rietveld fit of laboratory powder X-ray diffraction data of compound **2**. The calculated pattern is shown in light red, the experimental data in dark red and the difference in grey. 8
6 Rietveld fit of laboratory powder X-ray diffraction data of compound **4**. The calculated pattern is shown in light green, the experimental data in dark green and the difference in grey. 8
7 DFT optimised models of orderings in **2** viewed along the *c* axis 12
8 Symmetry analysis of the resulting spacegroup derived from the combination of conventional octahedral tilts with a complex conventional tilt with periodicity of four ($[\frac{1}{2}\frac{1}{2}\frac{1}{4}]T_2$ irrep) along a single axis in the presence of rocksalt M site order (R_2^+ irrep.). Structures with two tilts, which are not feasible in NCS-perovskites have a grey background. Non-centrosymmetric spacegroups are shown in bold. 12
9 Venn diagram showing the secondary order parameters deriving from the given modes in the $P\bar{4}2c$ structure. Note that Jahn-Teller distortions on alternating M-sites (R_3^- irrep) could stabilise the desired complex T_2 tilt in the presence of rocksalt M-site order. . . 13

List of Tables

1 Single crystal experimental details for compounds **1** and **2** 4
2 Single crystal experimental details for compounds **3**, **4** and Gua(SCN) 5
3 Rietveld refinement fit metrics for **3** refined using the SCXD model against synchrotron powder X-ray diffraction data 9
4 DFT-optimised unit cell parameters for models of compound **2** with seven different K^+ orderings, and the DFT-optimised experimental structure 11
5 DFT derived energy for compound **3** for the SCXD derived model and models with inverted $MeNH_3^+$ cations 11

1 Synthesis

1.1 Synthesis of 2 K{Ni[Bi(SCN)$_6$]}

KSCN (6 mmol, 0.583 g) was added to a stirred suspension of $Bi(NO_3) \cdot 5\,H_2O$ (1 mmol, 0.485 g) in 10 mL of butanone, producing a yellow-orange solution and a white precipitate of KNO_3. $Ni(NO_3)_2 \cdot 6H_2O$(1 mmol, 0.290 g), was then added producing a dark green solution. The reaction mixture was stirred for overnight and then filtered to remove KNO_3, yielding a dark green solution of KNi[Bi(SCN)$_6$]. This solution was then left to slowly evaporate on a covered watchglass, yield dark red single crystals of KNi[Bi(SCN)$_6$].
Compounds **1**, **3** and **4** were synthesised using an analogous routes, where KSCN was substituted for the appropriate thiocyanate salt.

2 X-ray Diffraction Measurements

2.1 Single Crystal X-ray Diffraction

Single crystals were selected and mounted using perfluoropolyether oil on a polymer-tipped MiTeGen MicroMountTM and cooled rapidly to 120 K or 180 K in a stream of cold N_2 using an Oxford Cryosystems open flow cryostat.[1] Single crystal X-ray diffraction data were collected for compound **1** using a Nonius KappaCCD diffractometer, using graphite monochromated MoKα radiation (λ = 0.7107 Å) at 180 K. Data for compound **2** were collected on an Oxford Diffraction GV1000 (TitanS2 CCD area detector, mirror-monochromated Cu-Kα radiation source; λ = 1.54184 Å, ω scans) at 120 K. Data for compound **3** were collected on an Oxford Diffraction GV1000 (AtlasS2 CCD area detector, mirror-monochromated Cu-Kα radiation source; λ = 1.54184 Å, ω scans) at 120 K. Data for compound **4** were collected in Experiments Hutch 1 (EH1) of Beamline I19, at Diamond Light Source.[2] The data were collected at a wavelength of 0.6889 Å on a Fluid Film Devices 3-circle fixed-chi diffractometer using a Dectris Pilatus 2M detector. Data for Gua(SCN) were collected on an Oxford Diffraction SuperNova Duo diffractometer (Atlas CCD area detector, mirror-monochromated Cu-Kα radiation source; λ = 1.54184 Å, ω scans).
Cell parameters were refined from the observed positions of all strong reflections. Integration and absorption corrections were carried out for laboratory data using a Gaussian numerical method with beam profile correction (CrysAlisPro)[3] and for synchrotron data using the xia2[4] and DIALS software packages[5]. Structures were solved within Olex2[6] by dual space iterative methods (SHELXT)[7] and all non-hydrogen atoms refined by full-matrix least-squares on all unique F^2 values with anisotropic displacement parameters (SHELXL).[8] Hydrogen atoms in compound **2** and in Gua(SCN) were located in the difference map and refined with constrained geometries and riding thermal parameters. H atoms could not be reliably located or positioned in compounds **3** and **4** due to structural disorder and so were omitted. Structures were checked with checkCIF (https://checkcif.iucr.org/). CCDC-2045274-8 contain the supplementary data for these compounds. These data can be obtained free of charge from The Cambridge Crystallographic Data Centre via www.ccdc.cam.ac.uk/data_request/cif.

2.2 Single Crystal Neutron Diffraction

Monochromatic single crystal diffraction data were collected on the four-circle D19 diffractometer at ILL (Grenoble, France) with Cu 331 monochromated radiation (take-off angle $2\theta_M$ = 69.9°), providing neutrons with a wavelength of 0.94558 Å, which is a good compromise between instrumental resolution, data completeness and the overlapping of neighbouring reflections. The sample was placed in a closed-circuit displex cooling device, which was operated following a ramp of 2 K min^{-1}. The measurement strategy consisted of several ω scans (25 at 20 K and 13 at 260 K) with steps of 0.07° at different χ and ϕ positions. Unit-cell determinations were performed using PFIND and DIRAX programs, and processing of the raw data was applied using RETREAT and RAFD19 programs.[9–11] Structures were refined within Olex2[6] using SHELXL[8]. All atoms were refined by full-matrix least-squares on all unique F^2 values with anisotropic displacement parameters. CCDC-2045258-9 con-

tain the supplementary data for these compounds. These data can be obtained free of charge from The Cambridge Crystallographic Data Centre via www.ccdc.cam.ac.uk/data_request/cif.

Table 1: Single crystal experimental details for compounds **1** and **2**

	**1** 20N	**1** 120X	**1** 260N	**2**
Empirical formula	C6 H4 Bi1 N4 N1 S3	C6 H4 Bi1 N4 N1 S3	C6 H4 Bi1 N4 N1 S3	C6 Bi1 K1 N6 Ni1 S6
Formula weight (Da)	634.21	634.21	634.21	655.27
T (K)	20	120	260	180
λ (Å)	0.94558	1.54184	0.94558	0.71073
Radiation	Neutron	Cu Kα	Neutron	MoKα
Crystal system	Triclinic	Triclinic	Triclinic	Triclinic
Space group	$P\overline{1}$	$P\overline{1}$	$P\overline{1}$	$P\overline{1}$
a (Å)	8.44100(10)	8.4699(2)	8.4472(12)	8.5073(2)
b (Å)	8.68990(10)	8.6913(2)	8.7119(10)	8.5686(2)
c (Å)	11.8177(2)	11.9058(3)	11.9671(9)	11.9651(3)
α °	97.5740(10)	97.187(2)	97.041(6)	97.5730(10)
β °	92.1370(10)	91.149(2)	90.364(7)	89.7810(10)
γ °	91.8170(10)	91.610(2)	91.558(9)	90.9970(10)
V (Å^3)	858.10(2)	868.96(4)	873.67(17)	864.46(4)
Z	2	2	2	2
ρ_{calc} (g cm^{-3})	2.455	2.424	2.411	2.517
μ (mm^{-1})	0.04734	27.765	0.04734	12.215
$F(000)$	592	592	592	608
Crystal size (mm)	2.9 × 2.5 × 2.2	0.247 × 0.241 × 0.087	2.9 × 2.5 × 2.2	0.06 × 0.05 × 0.02
θ range (°)	4.585–60.791	3.743–73.681	4.552–60.723	1.717–27.508
Reflections Measured	18745	15829	9536	7203
Independent Reflections	9590	3468	6293	3926
Completeness / max θ (°)	0.848 / 60.791	0.988 / 73.681	0.548 / 60.723	0.992 / 27.508
Absorption correction	empirical	gaussian	empirical	multi-scan
Transmission max. / min.	0.9203/0.8824	0.591/0.101	0.9203/0.8824	1.0 / 0.83
Refinement Method	F^2	F^2	F^2	F^2
Data/ restraints/parameters	9590/ 0/ 232	3468 / 10 / 212	6293 / 0 / 233	7203 / 0 /196
Goodness-of-fit on F^2	1.151	1.096	1.266	1.075
Final $R(F)$ $(I > 2\sigma)$	0.0448	0.0228	0.0963	0.0568
$wR2(F^2)$ (all data)	0.1117	0.0652	0.3111	0.1452
Max diff. peak/hole (e^-/A^3)	$+1.227$ / -1.752	$+1.621$ / -1.218	$+2.564$ / -2.165	$+3.567$/-3.225
CCDC code	2045258	2045274	2045259	2045276

Table 2: Single crystal experimental details for compounds **3**, **4** and Gua(SCN)

	**3**	**4**	Gua(SCN)
Empirical formula	C7 Bi1 N7 Ni1 S6	C0.92 Bi0.15 N1.25 Ni0.17 S0.86	C2 H6 N4 S
Formula weight (Da)	642.19	97.32	118.17
T (K)	120	100	120
λ (Å)	1.54184	0.6889	1.54184
Radiation	Cu Kα	Synchrotron X-ray	Cu Kα
Crystal system	Monoclinic	Cubic	Triclinic
Space group	$P1\frac{2}{n}1$	$Pn\overline{3}$	$P\overline{1}$
a (Å)	12.21600(10)	12.12528(2)	6.9352(6)
b (Å)	36.8375(4)	12.12528(2)	7.0096(5)
c (Å)	24.5263(3)	12.12528(2)	12.3675(7)
α °	90	90	90.894(5)
β °	90.2240(10)	90	104.311(7)
γ °	90	90	101.787(7)
V (Å^3)	11036.9(2)	1782.689(10)	568.89(7)
Z	24	24	4
ρ_{calc} (g cm^{-3})	2.319	2.176	1.38
μ mm$^{-}1$	26.251	9.725	4.11
$F(000)$	7152	1084	248
Crystal size (mm)	0.065 × 0.057 × 0.022	0.09 × 0.06 × 0.02	0.206 × 0.115 × 0.047
θ range (°)	3.600–67.684	2.302–35.897	3.697–73.681
Reflections Measured	123648	36116	2392
Independent Reflections	21661	1524	2256
Completeness / max θ (°)	0.982 / 67.684	0.979 / 35.897	0.982 / 73.681
Absorption correction	gaussian	empirical	gaussian
Transmission max. / min.	1.0 /0.937	1.0 / 0.9719	1.00 / 0.854
Refinement Method	F^2	F^2	F^2
Data/ restraints/parameters	21661 / 0 / 1272	1524 / 0 / 45	2256 / 0 /127
Goodness-of-fit on F^2	1.114	1.043	1.126
Final $R(F)$ ($I > 2\sigma$)	0.0422	0.0263	0.0898
$wR2(F^2)$ (all data)	0.0962	0.0866	0.2627
Max diff. peak/hole (e^-/A^3)	$+1.510/-1.895$	$+0.619/-0.507$	$+0.905/-0.539$
CCDC code	2045278	2045277	2045275

2.3 Powder X-ray Diffraction

A high-resolution synchrotron X-ray powder diffraction measurement of **3**, $MeNH_3\{Ni[Bi(SCN)_6]\}$, was carried out at beamline I11 at the Diamond Light Source (APS) using a wavelength of 0.82659 Å. The sample was loaded into a 0.5 mm diameter glass capillary. Rietveld refinement of the data was carried out using Topas Academic 6.[12,13] Lattice parameters were allowed to refine freely, along with the crystallographic size and strain. Atomic coordinates and atomic displacement parameters were fixed to those determined from single-crystal diffraction. H-atoms were omitted. The superlattice reflections are weak because they are primarily due to the light atoms in the frameworks ($MeNH^+$), NCS^-) rather than the heavy metal atoms. Refinement statistics are included in Tbl. 3. Laboratory powder X-ray diffraction measurements of **2**, $K\{Ni[Bi(SCN)_6]\}$, and **4**, $Gua_3\{Ni_6[Bi(SCN)_6]_5\}$, were carried out using a Panalytical Empyrean diffractometer using CuK$\alpha1$ radiation $\lambda = 1.540562$ (**2**),

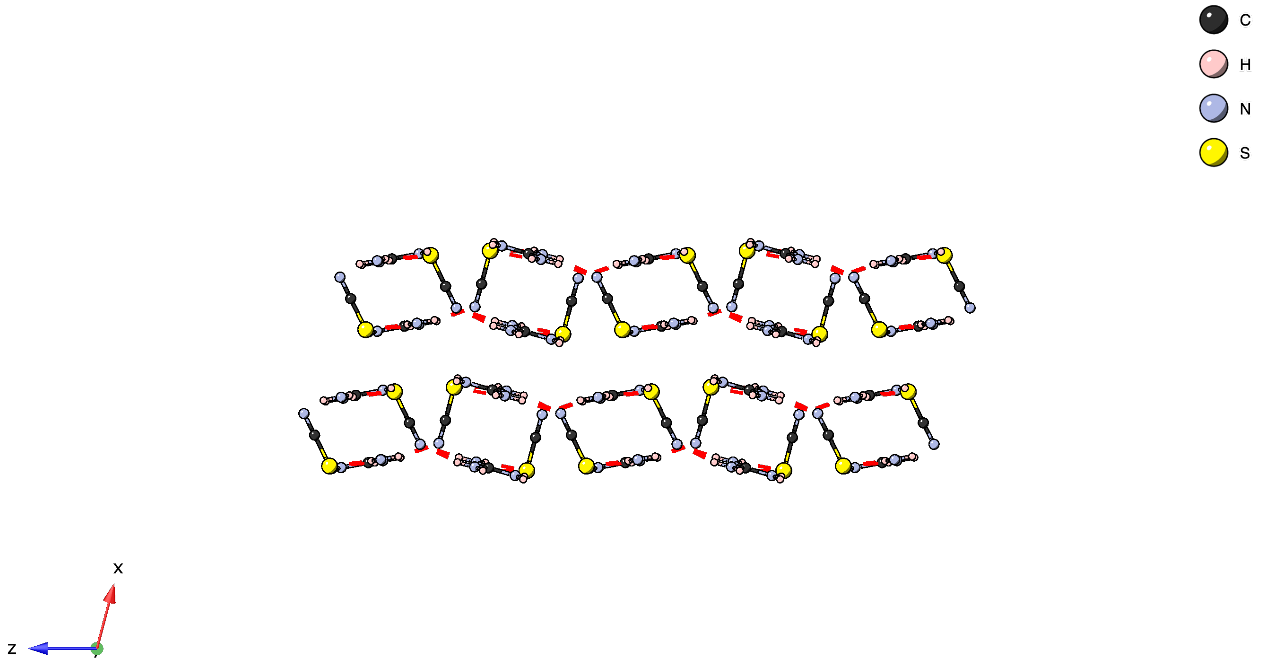

Figure 1: Gua(SCN) viewed along b

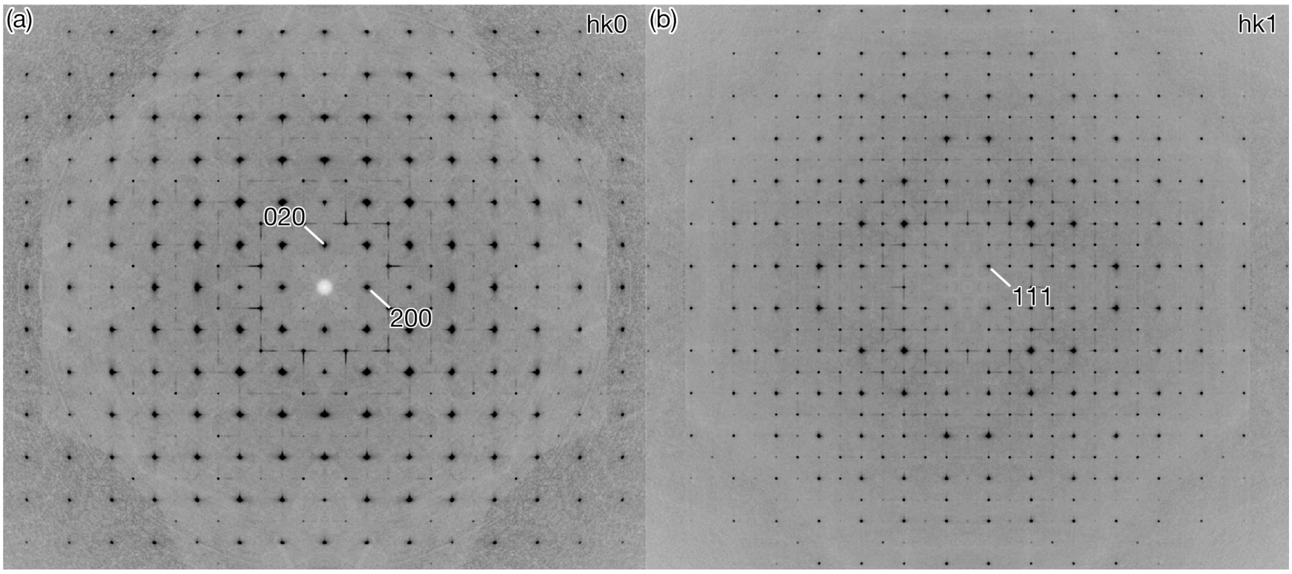

Figure 2: Diffuse scattering in **4** with symmetry averaging applied ($m\bar{3}$)

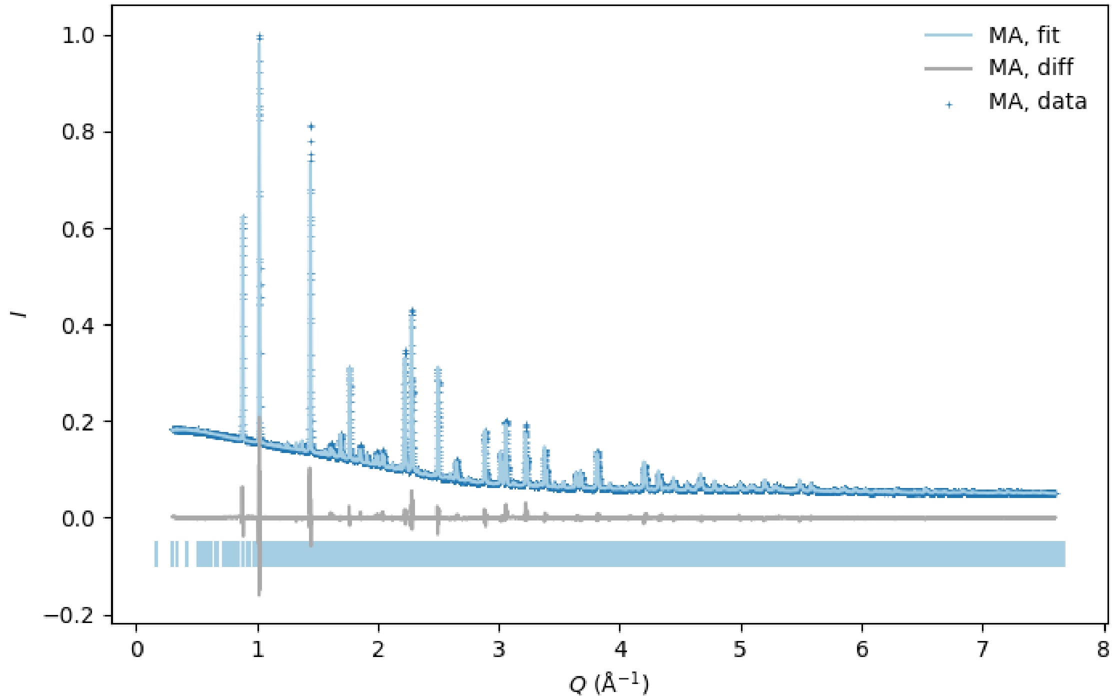

Figure 3: Rietveld fit of synchrotron powder X-ray diffraction data of compound **3**. The calculated pattern is shown in light blue, the experimental data in dark blue and the difference in grey.

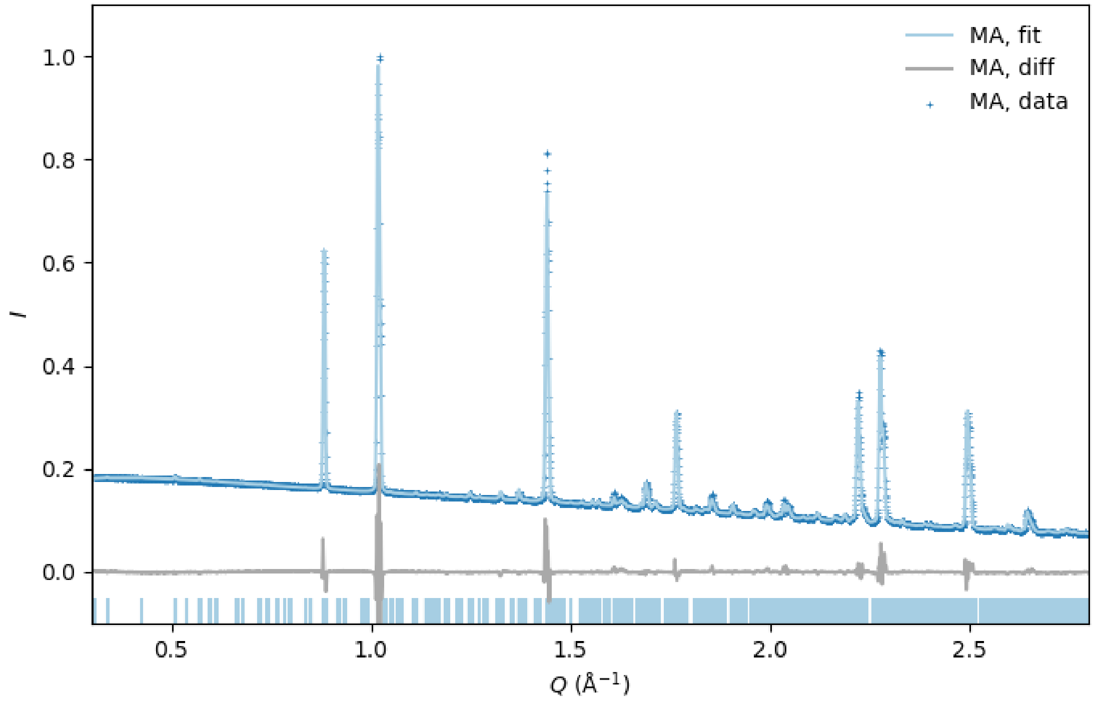

Figure 4: Rietveld fit of synchrotron powder X-ray diffraction data of compound **3**, zoomed in on low Q region. The calculated pattern is shown in light blue, the experimental data in dark blue and the difference in grey.

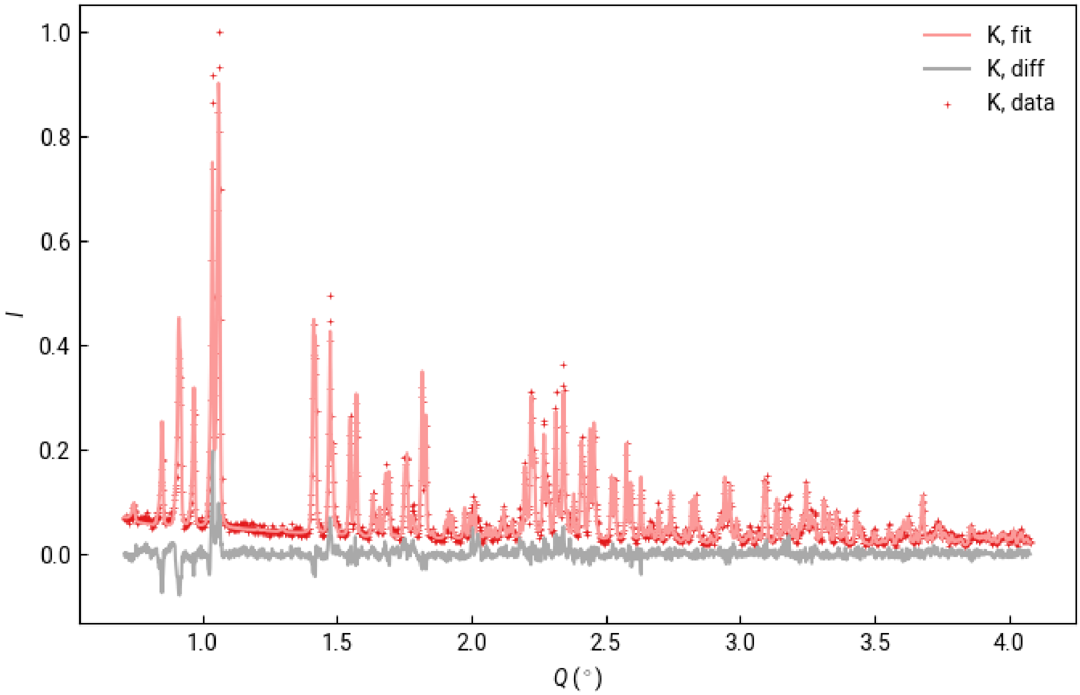

Figure 5: Rietveld fit of laboratory powder X-ray diffraction data of compound **2.** The calculated pattern is shown in light red, the experimental data in dark red and the difference in grey.

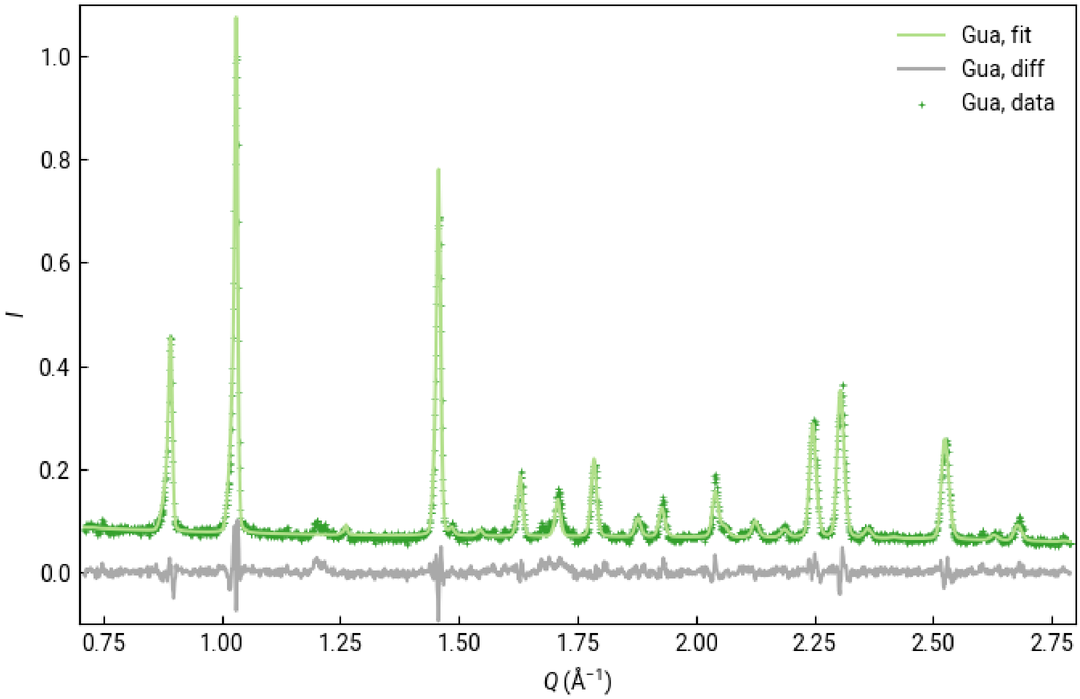

Figure 6: Rietveld fit of laboratory powder X-ray diffraction data of compound **4.** The calculated pattern is shown in light green, the experimental data in dark green and the difference in grey.

CuKα radiation $\lambda = 1.5420$ (**4**), in flat plate geometry. Rietveld refinement of the data was carried out using Topas Academic 6.[12,13] Lattice parameters were allowed to refine freely, along with the crystallographic size and strain. Atomic coordinates and atomic displacement parameters were fixed to those determined from single-crystal diffraction. H-atoms were omitted.

Table 3: Rietveld refinement fit metrics for **3** refined using the SCXD model against synchrotron powder X-ray diffraction data

R_{wp}	2.976
R_p	1.438
R_{Bragg}	2.080
S	9.648

3 Tolerance Factor Calculations

The tolerance factor for atomic frameworks is defined as $\tau = \frac{r_A + r_X}{\sqrt{2}(r_M + r_x)}$, where r_A is the radius of the A-site cation, r_M the radius of the M-site cation, r_x is the radius of the X-site anion. In molecular frameworks, an effective radius can be defined for (alkyl)ammonium cations, but the extended nature of anions means they are often better modelled by a cylinder, as proposed by Kieslich, Sun and Cheetham (KSC).[14] Each anion therefore has two, a radius and a length:
$\tau = \frac{r_A + r_X}{\sqrt{2}(r_M + \frac{l_x}{2})}$,
where r_A is the effective radius of the A-site cation, r_M the radius of the M-site cation, r_x is the effective radius of the X-site anion, and l_x the effective length of the X-site anion. Using the library of existing NCS-perovskites we estimated the effective parameters of the NCS^- ligands to be $r_{\mathrm{NCS}} = 2.00$ Å, $l_{\mathrm{NCS}} = 4.34$ Å, comparable to previous estimates of spherical radii for NCS^-.[15] We used radii derived by Shannon for atomic cations,[16] and those derived by KSC for molecular cations.[14] For mixed cation perovskites the mean of the two radii was used to calculate τ.

4 Density Functional Theory Calculations

We performed density functional theory (DFT) calculations to probe the structures and energetics of several compounds considered in this study.
For compounds **2** and **3**, cell optimizations were performed using the CP2K code (version 6.0), which uses a mixed Gaussian/plane-wave basis set.[17,18] We employed double-ζ polarization quality Gaussian basis sets[19] and a 400 Ry plane-wave cutoff for the auxiliary grid, in conjunction with the Goedecker-Teter-Hutter pseudopotentials[20,21]. A convergence threshold of 1.0×10^{-6} Hartree was used for the self-consistent field cycle; cell optimizations (including cell parameters and atomic positions) were considered to be converged when the maximum force on atoms fell below 4.5×10^{-4} Hartree/Bohr. Spin-polarization was needed to describe open-shell Ni^{2+} cations, and we assume a high-spin ferromagnetic solution for all Ni^{2+} cations in our calculations. All DFT calculations were performed in the Γ-point approximation, using the PBE functional,[22] with Grimme's D3 van der Waals correction (PBE+D3)[23].
For $Cs_3\{Fe[Bi(SCN)_6]\}_4$ a spin-polarized DFT+U method (with Grimme's D3 van der Waals correction[23]) was employed to describe better the two different oxidation states of Fe in this compound (Fe^{2+} and Fe^{3+}, using the Vienna Ab initio Simulation Package (VASP 5.4.4)[24]. In our DFT+U calculations, we used $U = 3.0$ eV for the d-electrons of Fe^{2+} cations and $U = 6.0$ eV for the d-electrons of Fe^{3+} cations and a high-spin ferromagnetic solution was assumed for all Fe^{2+} and Fe^{3+} cations. We used a plane-wave basis set with a kinetic energy cutoff of 400 eV to expand the wave functions. The Perdew-Burke-Ernzerhof functional[22] in combination with the projector augmented wave method[25,26] were used to solve the Kohn-Sham equations. An energy convergence threshold of 10^{-4} eV was used for all total energy calculations. Structural optimizations, which included optimisation of cell parameters and atomic positions, were considered converged once all interatomic forces fell below 0.02 eV/Å. All DFT calculations were performed in the Γ-point approximation for sufficiently large cells.
Starting configurations for investigating the different orderings of $K\{Ni[Bi(SCN)_6]\}$ were created by first generating a ($\sqrt{2} \times \sqrt{2} \times 1$) supercell of the experimental $Fe[Bi(SCN_6)]$ structure, and then placing four K^+ cations into the centre of the pseudocubic cages with each of the different orderings. The close similarity between the optimised structure with columnar [001] A-site order generated with this method and the experimental structure therefore shows the reliability of this approach. The anisotropy of the structures was asssesed by calculating the strain relative to a hypothetical cubic cell, where $\alpha = \beta = \gamma = 90°$ and $a_{\mathrm{cubic}} = V^{\frac{1}{3}}$. The principal strains (eigenvalues of the strain matrix) are reported in Tbl. 4.[27]

Table 4: DFT-optimised unit cell parameters for models of compound **2** with seven different K^+ orderings, and the DFT-optimised experimental structure

Model	*a* (Å)	*b* (Å)	*c* (Å)	α (°)	β (°)	γ (°)	V (Å^3)	a_{cubic} (Å)		strain	(%)
Expt.	12.3746	12.1038	12.0364	96.612	84.369	91.234	1782.08	12.1239	7.75	-0.01	7.77
Rocksalt	12.2034	12.2035	12.1930	89.579	89.582	88.184	1814.83	12.1977	-1.56	-0.19	1.75
Layer (100)	12.1448	12.2389	12.2354	90.465	89.405	87.855	1817.22	12.2031	-2.22	0.44	1.78
Layer (010)	12.2408	12.1385	12.2388	89.418	90.469	87.801	1817.00	12.2026	-2.28	0.46	1.82
Layer (001)	12.2015	12.2018	12.3409	89.710	89.712	86.469	1833.79	12.2400	-3.48	0.76	2.73
Col [100]	12.2118	12.0950	12.2594	89.835	89.768	86.343	1807.03	12.1802	-3.54	0.62	2.92
Col [010]	12.0944	12.2112	12.2596	89.765	89.839	86.339	1806.87	12.1799	-3.55	0.63	2.92
Col [100]	12.0988	12.2700	12.1455	94.831	84.598	89.948	1788.57	12.1386	-6.62	0.48	6.14

Table 5: DFT derived energy for compound **3** for the SCXD derived model and models with inverted $MeNH_3^+$ cations

Config.	E_{total} (Hartree)	ΔE (kJ/mol per cell)	n($MeNH_3^+$)	ΔE (kJ/mol per $MeNH_3^+$)
Expt.	-8378.72661	0.0	-	0.0
MA-1	-8378.714436	32.0	2	16.0
MA-2	-8378.718684	20.8	2	10.4
MA-3	-8378.697917	75.3	4	18.8
MA-4	-8378.718645	20.9	2	10.5
MA-5	-8378.700874	67.6	4	16.9
MA-6	-8378.714088	32.9	2	16.4
MA-7	-8378.7094	45.2	4	11.3
MA-8	-8378.692082	90.7	4	22.7

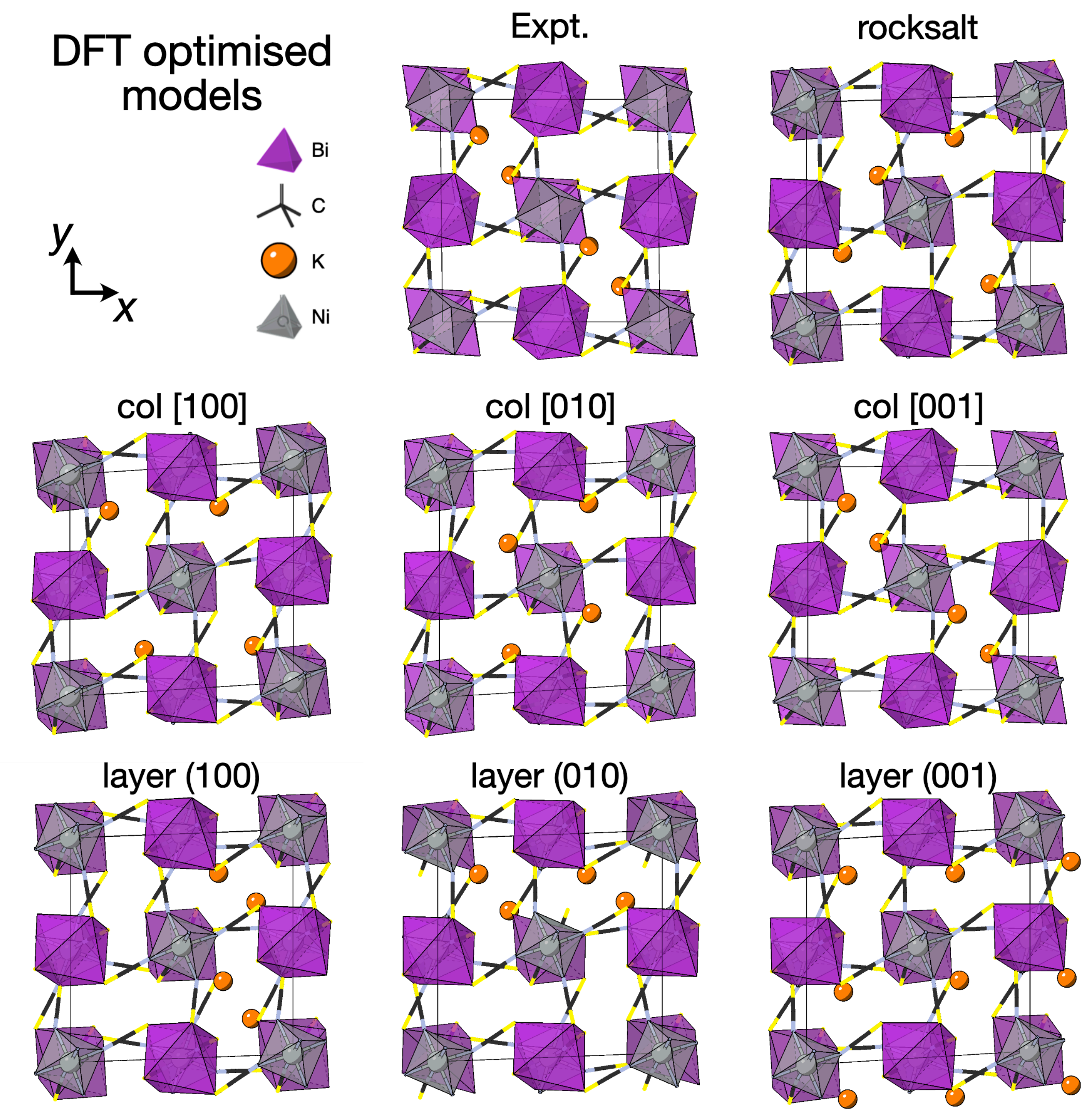

Figure 7: DFT optimised models of orderings in **2** viewed along the *c* axis

	**T_2(a,0;0,0;0,0)**	**T_2(a,a;0,0;0,0)**
**M_2^+(0;0;a)**	*Pban*	*Pnna*
**M_2^+(0;a;a)**	***$P\bar{4}2c$***	*Ccca*
**R_5^-(0,0,a)**	*C2/c*	*C2/c*
**R_5^-(0,a,a)**	*$P2_1/c$*	*P2/c*

Figure 8: Symmetry analysis of the resulting spacegroup derived from the combination of conventional octahedral tilts with a complex conventional tilt with periodicity of four ($[\frac{1}{2}\frac{1}{2}\frac{1}{4}]T_2$ irrep) along a single axis in the presence of rocksalt M site order (R_2^+ irrep.). Structures with two tilts, which are not feasible in NCS-perovskites have a grey background. Noncentrosymmetric spacegroups are shown in bold.

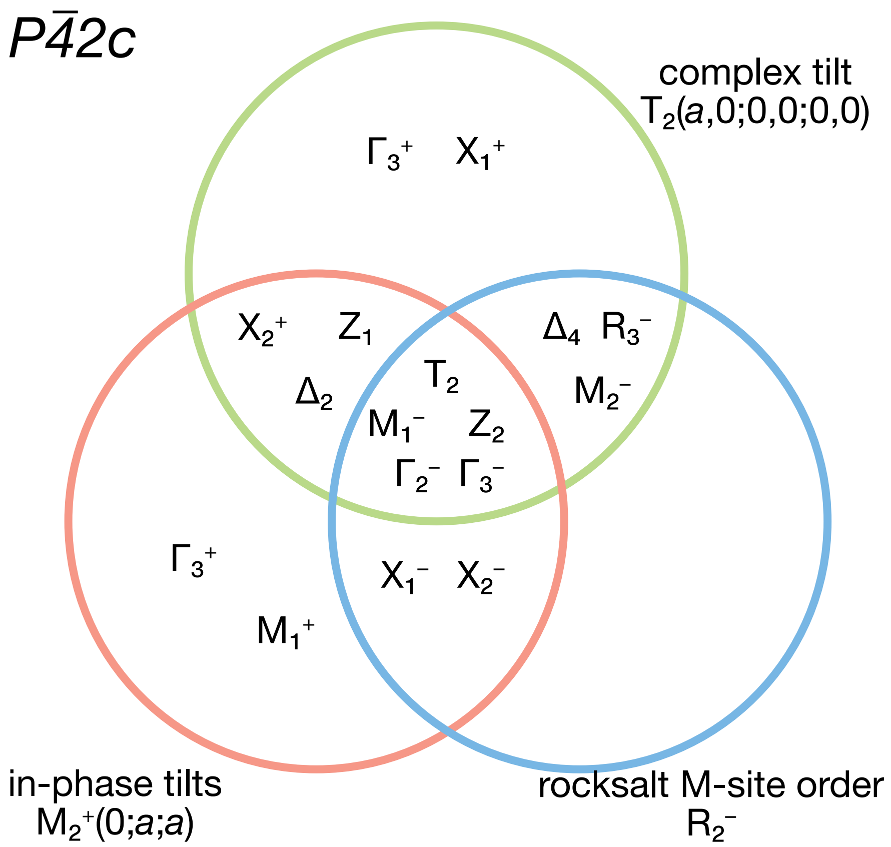

Figure 9: Venn diagram showing the secondary order parameters deriving from the given modes in the $P\bar{4}2c$ structure. Note that Jahn-Teller distortions on alternating M-sites (R_3^- irrep) could stabilise the desired complex T_2 tilt in the presence of rocksalt M-site order.

References

[1] J. Cosier *et al.*, *J. Appl. Crystallogr.*, 1986, **19**, 105–107.
[2] D. R. Allan *et al.*, *Crystals*, 2017, **7**, 336.
[3] *CrysAlisPro Software System, Version 1.171.40.45a*, Rigaku Corporation, 2018.
[4] G. Winter, *J Appl Cryst*, 2010, **43**, 186–190.
[5] G. Winter *et al.*, *Acta Cryst D*, 2018, **74**, 85–97.
[6] O. V. Dolomanov *et al.*, *J. Appl. Crystallogr.*, 2009, **42**, 339–341.
[7] G. M. Sheldrick, *Acta Crystallogr.*, 2015, **A71**, 3–8.
[8] G. M. Sheldrick, *Acta Crystallogr.*, 2015, **C71**, 3–8.
[9] A. J. M. Duisenberg, *J Appl Cryst*, 1992, **25**, 92–96.
[10] G. J. McIntyre *et al.*, *Acta Cryst A*, 1988, **44**, 257–262.
[11] C. Wilkinson *et al.*, *J Appl Cryst*, 1988, **21**, 471–478.
[12] H. Rietveld, *J. Appl. Crystallogr.*, 1969, **2**, 65–71.
[13] A. A. Coelho *et al.*, *Powder Diffr.*, 2011, **26**, S22–S25.
[14] G. Kieslich *et al.*, *Chem. Sci.*, 2015, **6**, 3430–3433.
[15] Y. Iwadate *et al.*, *J. Phys. Chem.*, 1982, **86**, 5205–5208.
[16] R. D. Shannon, *Acta Cryst*, 1976, **A32**, 751–767.
[17] J. VandeVondele *et al.*, *Comput. Phys. Commun.*, 2005, **167**, 103–128.
[18] J. Hutter *et al.*, *WIREs Comput. Mol. Sci.*, 2014, **4**, 15–25.
[19] J. VandeVondele *et al.*, *J. Chem. Phys.*, 2007, **127**, 114105.
[20] S. Goedecker *et al.*, *Phys. Rev. B*, 1996, **54**, 1703–1710.
[21] M. Krack, *Theor Chem Acc*, 2005, **114**, 145–152.
[22] J. P. Perdew *et al.*, *Phys. Rev. Lett.*, 1996, **77**, 3865–3868.
[23] S. Grimme *et al.*, *J. Chem. Phys.*, 2010, **132**, 154104.
[24] G. Kresse *et al.*, *Phys. Rev. B*, 1996, **54**, 11169–11186.
[25] P. E. Blöchl, *Phys. Rev. B*, 1994, **50**, 17953–17979.
[26] D. Hobbs *et al.*, *Phys. Rev. B*, 2000, **62**, 11556–11570.
[27] M. J. Cliffe *et al.*, *J. Appl. Crystallogr.*, 2012, **45**, 1321–1329.
